# Palbociclib Enhances Migration and Invasion of Cancer Cells via Senescence-Associated Secretory Phenotype-Related CCL5 in Non-Small-Cell Lung Cancer

**DOI:** 10.1155/2022/2260625

**Published:** 2022-09-27

**Authors:** Pengzhou Kong, Xin Yang, Yingying Zhang, Huiling Dong, Xiangchen Liu, Xiaoqin Xu, Xinri Zhang, Yiwei Shi, Mingxia Hou, Bin Song

**Affiliations:** ^1^Key Laboratory of Cellular Physiology of the Ministry of Education, & Department of Pathology, Shanxi Medical University, Taiyuan, Shanxi, China; ^2^Department of Respiratory and Critical Care Medicine, The First Hospital, Shanxi Medical University, Taiyuan, Shanxi, China; ^3^Department of Etiology, Shanxi Cancer Hospital, Taiyuan, Shanxi, China; ^4^Cancer Center, Shanxi Bethune Hospital, Shanxi Academy of Medical Sciences, Tongji Shanxi Hospital, Third Hospital of Shanxi Medical University, Shanxi, China

## Abstract

Palbociclib is the first CDK4/6 inhibitor approved by FDA and has been studied in many types of cancer. However, some studies showed that it could induce epithelial-mesenchymal transition (EMT) of cancer cells. To test the effect of palbociclib on non-small-cell lung cancer (NSCLC) cells, we treated NSCLC cells with different concentrations of palbociclib and detected its effects via MTT, migration and invasion assays, and apoptosis test. Further RNA sequencing was performed in the cells treated with 2 *μ*M palbociclib or control. And Gene Ontology, Kyoto Encyclopedia of Genes and Genomes (KEGG), Gene Set Enrichment Analysis (GSEA), and protein-protein interaction network (PPI) were analyzed to explore the mechanism of palbociclib. The results showed that palbociclib significantly inhibited the growth of NSCLC cells and promoted apoptosis of cells, however, enhanced the migration and invasion abilities of cancer cells. RNA sequencing showed that cell cycle, inflammation-/immunity-related signaling, cytokine-cytokine receptor interaction, and cell senescence pathways were involved in the process, and CCL5 was one of the significantly differential genes affected by palbociclib. Further experiments showed that blocking CCL5-related pathways could reverse the malignant phenotype induced by palbociclib. Our results revealed that palbociclib-induced invasion and migration might be due to senescence-associated secretory phenotype (SASP) rather than EMT and suggested that SASP could act as a potential target to potentiate the antitumor effects of palbociclib in cancer treatment.

## 1. Introduction

Lung cancer is one of the most common malignant tumors in both men and women, with high morbidity and mortality [1]. Small-cell lung cancer (SCLC) and non-small-cell lung cancer (NSCLC) are the two main histological types of lung cancer. The latter accounts for 85-90% of patients with lung cancer and mainly consists of lung squamous cell carcinoma (LUSC) and lung adenocarcinoma (LUAC). Despite the rapid developments in the diagnosis and treatment, lung cancer still has a poor prognosis, and its mortality still ranks the first in all malignant tumors [2]. Thus, it is urgent to find novel therapeutic targets for lung cancer.

One of the characteristics of tumor cells is the disorder of cell cycle regulation, which is due to sustaining proliferative signaling or evading growth suppressors, leading to uncontrolled cell growth and proliferation [3]. Cyclin-dependent kinases (CDKs) play essential and important roles in regulating cell cycle phases in cell cycle progression and often are amplified or overexpressed in cancer tissues. Thus, they are ideal targets for cancer treatment [4]. A series of CDK inhibitors targeting specific CDKs have been developed and applied into clinical trials [5]. Palbociclib is the first CDK4/6 inhibitor approved by Food and Drug Administration (FDA) of U.S. and implied in metastatic ER^+^/HER2^−^ breast cancer. Research evidence has suggested that palbociclib blocks p16–CDK4/6–cyclin D1–RB signaling and inhibits aggressive growth, induces apoptosis, and represses invasion and metastasis of cancer cells [6]. It is also being reported in other types of cancer [7]. In most studies, palbociclib was also found to inhibit migration, invasion, and metastasis of cancer cells via blocking epithelial-mesenchymal transition (EMT) process in cancer [8, 9]. Only one study showed that anti-CDK4/6 therapy could induce EMT in pancreatic cancer cells which depended on TGF*β* signaling [10].

To identify the effect of palbociclib on NSCLC cells, we treated NSCLC cells with palbociclib of gradient concentration in the present study. We found that palbociclib treatment significantly inhibited the growth of NSCLC cells but also promoted their migration and invasion ability. Furthermore, transcriptome analysis showed that palbociclib induced cell cycle arrest and cell senescence. Senescence-associated secretory phenotype (SASP) and upregulated chemokine (C-C motif) ligand 5 (CCL5) expression could partly explain the enhancement of migration and invasion ability of NSCLC cells. Our results provided a useful clue for understanding the mechanism and potentiated the antitumor effects of palbociclib in cancer treatment.

## 2. Methods

### 2.1. Cell Culture

Lung cancer cell lines NCI-H226 (with LUSC) and NCI-H1650 (with LUAD) were purchased from Cell Bank of Type Culture Collection of Chinese Academy of Sciences (Shanghai, China). The cells were grown in RPMI-1640 (Gibco, Sigma-Aldrich, USA) supplemented with 10% fetal bovine serum (FBS, Gibco, Sigma-Aldrich, USA) and 1% penicillin-streptomycin solution (Biological Industries, Israel) at 37°C, 5% CO_2_.

### 2.2. Drug Treatment

Palbociclib was purchased from MedChemExpress (MCE, CA, USA) [11]. Ten milligrams of palbociclib was dissolved in 4.1323 ml sterilized triple-distilled water (concentration: 5 mmol/ml) and then was subpacked into 1.5 ml EP tubes and stored at -80°C. When used, the mother liquid was diluted with complete medium to 1 *μΜ* and 2 *μ*M, respectively. Antagonists of CCR5, TAK-779, was purchased from MCE (CA, USA).

### 2.3. Conditioned Media Collection

Conditioned media (CM) was collected according to references [12, 13]. Briefly, after 72 hours of treatment with 2 *μ*M palbociclib or control, the cells were washed twice with PBS to remove remnants of FBS and cultured in fresh RPMI-1640 without FBS for 24 hours at 37°C, 5% CO_2_. Then, the resultant CM was collected, centrifuged for 10 minutes at 1000 rpm and 4°C to remove cell and cellular debris, and frozen at -80°C for conditioned media study.

### 2.4. MTT Assay

Effect of palbociclib on cancer cell viability was determined using MTT assay according to the previous study [14]. Briefly, NSCLC cells were plated into 96-well plates at a density of 5 × 10^3^/well with five replications and incubated in normal conditions. After drug treatment for 72 hours, 20 *μ*l of 5 mg/ml of MTT solution was added in medium and incubated for 4 hr at 37°C. Then, the medium was discarded, and the crystals were dissolved by 150 *μ*l of DMSO. The final absorbance was measured at 490 nm to calculate the cell viability at different drug concentrations. The experiment was repeated at least three times.

### 2.5. Apoptosis Assay

The effect of palbociclib on cancer cell apoptosis was tested using Annexin V/propidium iodide (PI) staining method[15]. Briefly, the cells were treated with 2 *μ*M palbociclib or control for 72 h and collected by centrifugation. Then, the cells were resuspended in 500 *μ*l of 1×Annexin V binding buffer and incubated with Annexin V-FITC and PI for 5 min in the dark condition. At last, the cells were analyzed via flow cytometry. The experiment was repeated at least three times.

### 2.6. Cell Migration and Invasion Assay

After 72 hours of treatment with 2 *μ*M palbociclib or control, cell invasion and migration were examined by transwell assays according to the protocol of our previous study [16]. At the same time, NSCLC cells were treated by CM from palbociclib-treated H226 and/or H1650 cells, and cell invasion and migration were examined by transwell assay. To explore the effect of TAK-779 (selective antagonist of CCR5 and CXCR3), the drugs were added in the medium in the upper chambers to block the specific targets. Three replicates were set for each group, and each experiment was repeated at least three times.

### 2.7. Western Blot

Protein expression of SASP-related genes was detected through western blot. Briefly, after 72 hours of treatment with 2 *μ*M palbociclib, the cells were lysed, and proteins were collected and determined by the Bradford method. 30 *μ*g of total protein was loaded and separated by SDS-polyacrylamide gel electrophoresis and then transferred onto PVDF membranes. Blocked with 5% nonfat milk for 2 hours, the membranes were incubated with the special primary antibodies at 4°C overnight, with the horseradish peroxidase- (HRP-) labeled second antibodies for 1 hour at room temperature and then detected with a chemiluminescence reagent (TransGen Biotech, China). The relative amount of gene product was normalized to GAPDH. The western blot experiment was repeated three times at least. The antibodies used in this experiment are shown in Supplementary Table 1.

### 2.8. Enzyme-Linked Immunosorbent Assay

As a chemokine, CCL5 is produced and secreted from senescent aged cells [17]. Thus, the secreted CCL5 levels in the media of palbociclib-treated cells and the control cells were assessed by enzyme-linked immunosorbent assay (ELISA) using CCL5 ELISA kit (EK0494, Boster Biological Technology, CA, USA) according to the manual instruction.

### 2.9. Transcriptome-Sequencing and Bioinformatics Analysis

Transcriptome change induced by palbociclib was detected by RNA sequencing. After 24 hours of treatment with 2 *μ*M palbociclib, H226 and H1650 cells of the control group and treated group were collected for RNA sequencing by the Beijing Genomics Institute (BGI, China). The data was analyzed using Dr. Tom system (BGI, China) according to previous studies about Dr. Tom [18]. Significant differentially expressed genes (DEG, fold change ≥ twofold as well as a false discovery rate (FDR) ≤ 0.001) were screened between two groups in every cell line and compared gene lists using Venn diagrams. The shared genes were analyzed for the enriched pathway and function using the Gene Ontology (GO) and Kyoto Encyclopedia of Genes and Genomes (KEGG). Furthermore, Gene Set Enrichment Analysis (GSEA) and protein-protein interaction network (PPI) were analyzed to explore the mechanism of palbociclib.

### 2.10. Real-Time Quantitative PCR

Real-time quantitative PCR (RT-qPCR) was used to measure mRNA expression levels of CCL5 induced by palbociclib. Briefly, total RNA was extracted using RNAiso Plus (Takara, Bio Inc., Japan), and 2 *μ*g RNA was used to synthesize cDNA by the PrimeScript 1st strand cDNA Synthesis Kit (Takara, Bio Inc., Japan). Then, RT-qPCR was performed to detect the threshold cycle (Ct) using the SYBR Green method. The relative expression of CCL5 was determined by normalization to GAPDH expression and calculated using the 2^-*ΔΔ*Ct^ formula [19, 20]. All experiments were done in triplicate and repeated three times at least. The primers for genes were shown in Supplementary Table 1.

### 2.11. Senescence-Associated *β*-Galactosidase Staining

Senescence-associated *β*-galactosidase (SA-*β*-Gal) is highly correlated with senescent cells and tissues, and SA-*β*-Gal staining is a classical method to identify the presence of senescent cells [21]. Thus, we detected senescent cells induced by palbociclib using *β*-galactosidase staining kit (Beyotime, China) following the instruction. Briefly, after 72 hr of treatment with palbociclib, the cells were washed with PBS and fixed for 15 min at room temperature. Then, the cells were incubated with staining solution at 37°C overnight. Lastly, the cells were observed, and senescent cells stained with blue were counted.

### 2.12. Statistical Analysis

Experiment data were presented as mean ± SEM. The means were compared using Student's *t*-test between the two groups, and data from more than two groups were analyzed by one-way ANOVA. Correlation between genes was performed using nonparametric correlation (Spearman). *P* < 0.05 was considered statistically significant.

## 3. Results

### 3.1. Palbociclib Inhibited the Growth but Promoted the Migration and Invasion Ability of Lung Cancer Cells

**Figure 1:**
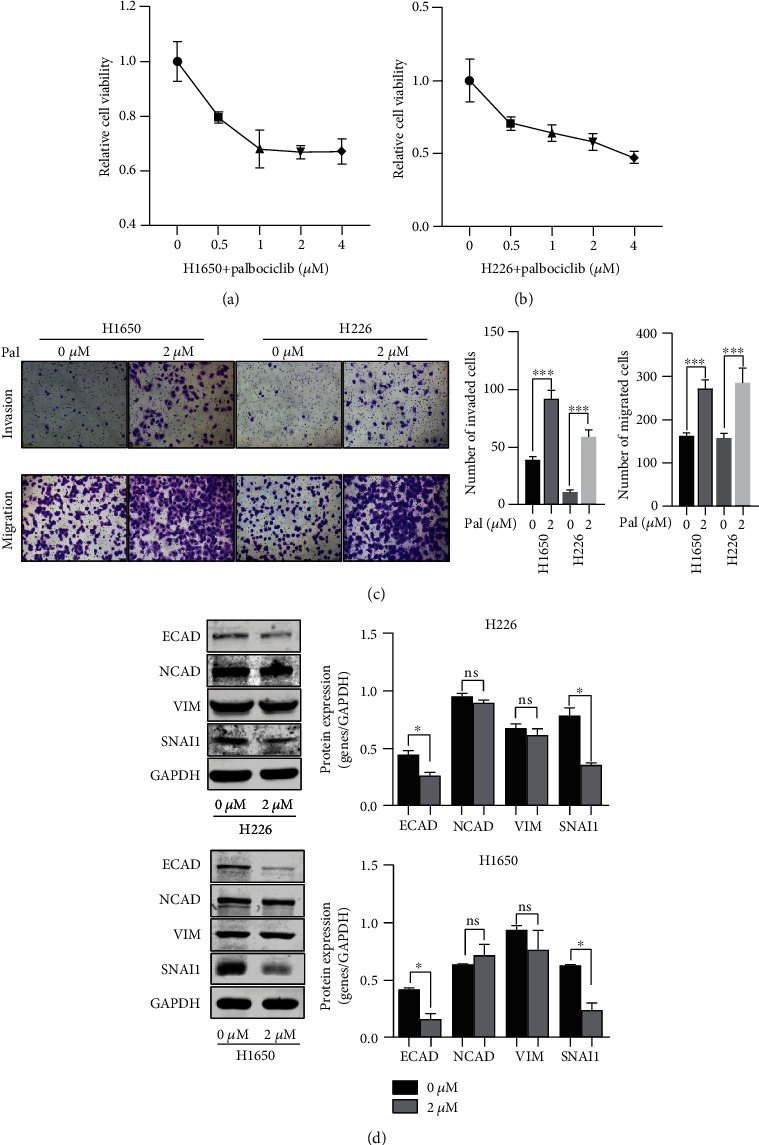
Effect of palbociclib on H1650 and H226 cells. (a and b) Effects of different concentrations of palbociclib on cell viability in H1650 and H226 cells, respectively. (c) Effect of palbociclib on cell migration and invasion in H1650 and H226 cells. Left: images of invaded and migrated H1650 and H226 cells treated with/without palbociclib. Scale bars represent 100 *μ*m. Right: the bar charts display the calculated numbers of invaded cells and migrated cells. (d) EMT-related markers' expression after palbociclib treatment. Left: gels of western blot for EMT-related markers' expression, with GAPDH as a loading control. Right: the bar charts display the relative expression of target genes on protein level. All experiments were repeated at least three times. Western blot result of every target protein cropped from the same gel in the same experiment and the proteins with similar size came from different gels in the same experiment. ∗*P* < 0.05, ∗∗*P* < 0.01, and ∗∗∗*P* < 0.001; ns: no significance.

We detected the inhibitory effect of different concentrations of palbociclib on the two lung cancer cell lines firstly. As expected, palbociclib significantly inhibited the growth in H226 and H1650 cells and presented remarkable anticancer activity (Figures 1(a) and 1(b)). Consistent with the previous studies [22, 23], apoptosis assay showed palbociclib significantly induced cell apoptosis in H226 and H1650 cells (supplementary Figure 1a). Interestingly, transwell assays showed that palbociclib significantly promoted cancer cells migration and invasion (Figure 1(c)). Similarly, the CM from palbociclib-treated H226 and H1650 cells significantly promoted cell migration and invasion in untreated H226 and H1650 (supplementary Figures 1b and 1c). All results indicated that palbociclib might promote cancer migration and invasion in some cases.

### 3.2. Palbociclib Does Not Induce EMT in NSCLC Cancer Cells

EMT is a key process that promotes epithelial cancer cells to get the ability of migration and invasion [24–26]. In a previous study, palbociclib was reported to induce EMT in pancreatic cancer cells [10]. However, most studies showed that EMT process was inhibited by palbociclib. Thus, we detected the effect of palbociclib on EMT markers in H226 and H1650 cells. The results showed that ECAD, the epithelial marker, was significantly decreased by 2 *μ*M palbociclib. Unexpectedly, NCAD and VIM, two mesenchymal markers, were decreased slightly after treatment with 2 *μ*M palbociclib. Meanwhile, SNAI1, the key transcript factor in EMT process, was significantly decreased (Figure 1(d)). The results suggested that palbociclib-induced invasion and migration might not depend on EMT in NSCLC cells.

### 3.3. Transcriptome Change Induced by Palbociclib

**Figure 2:**
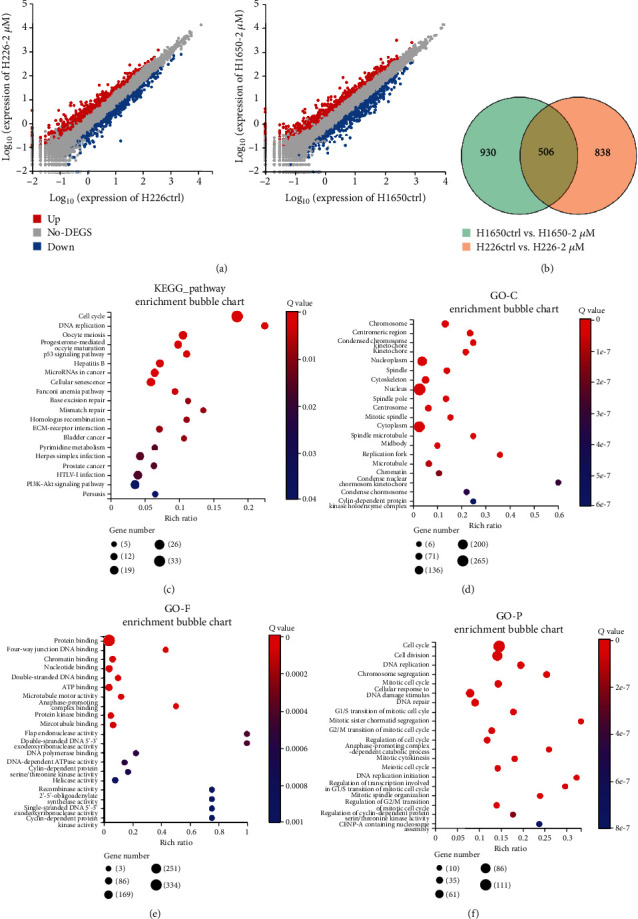
Transcriptome change induced by a palbociclib in NSCLC cells. (a) The diagram results showed the DEGs in H226 and H1650 cells after treatment with 2 *μ*M palbociclib. (b) The Venn diagram results showed that there were 506 shared DEGs in H226 and H1650 cells after treatment with 2 *μ*M palbociclib. (c) The KEGG analysis of DEGs induced by a palbociclib in NSCLC cells. (d) The GO analysis of DEGs induced by a palbociclib in NSCLC cells. (e) The GO analysis of DEGs induced by a palbociclib in NSCLC cells. (f) The GO analysis of DEGs induced by a palbociclib in NSCLC cells.

To explore the underlying mechanism of palbociclib-induced migration and invasion in NCSLC cells, we performed transcriptome sequencing in H226 and H1650 cells treated with 2 *μ*M palbociclib and analyzed the sequencing data using Dr. Tom system. We screen the DEGs in the two cell lines (Figure 2(a)) and compared them. The Venn diagram results showed that there were 506 shared DEGs in H226 and H1650 cells after treatment with 2 *μ*M palbociclib (Figure 2(b) and Supplementary Table 2).

**Figure 3:**
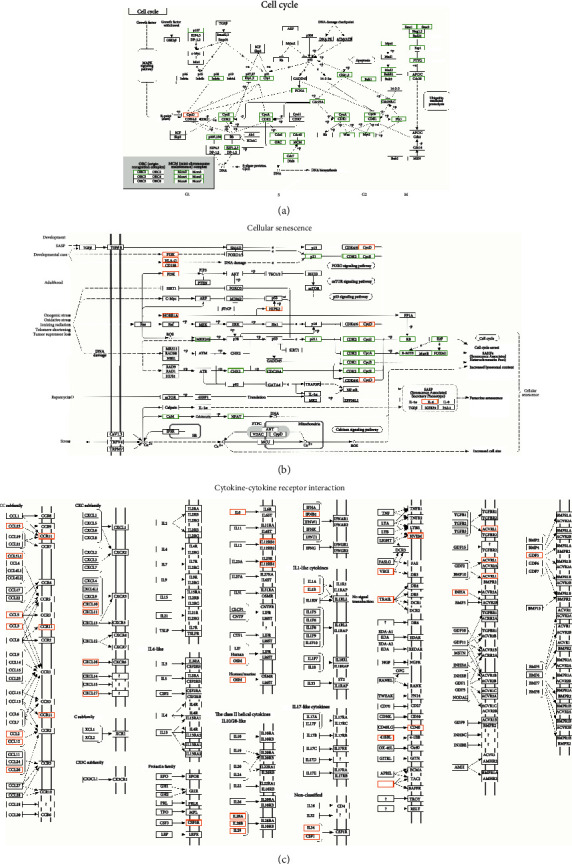
DEGs were enriched in cell cycle pathway (a), cell senescence (b), and cytokine-cytokine receptor interaction pathways (c).

Further KEGG (Figure 2(c), Supplementary Table 3) and GO (Figures 2(d)–2(f)) analyses of DEGs between treated cells and control cells showed that pathways related to cell cycle or DNA replication or meiosis were the most significant pathways affected by palbociclib, which is consistent with the previous reports. Then, we noticed that some DEGs were enriched in the pathways related to cell cycle or meiosis, infection/immune/inflammation, cell senescence, cytokine-cytokine receptor interaction, and so on (Figure 2(c)). In the above pathways, most genes involved in cell cycles were decreased (Figure 3(a)). In cell senescence pathway, the genes associated with cell cycles were downregulated and others were upregulated (Figure 3(b)). And genes coding cytokines and their receptors were significantly upregulated after palbociclib treatment (Figure 3(c)).

**Figure 4:**
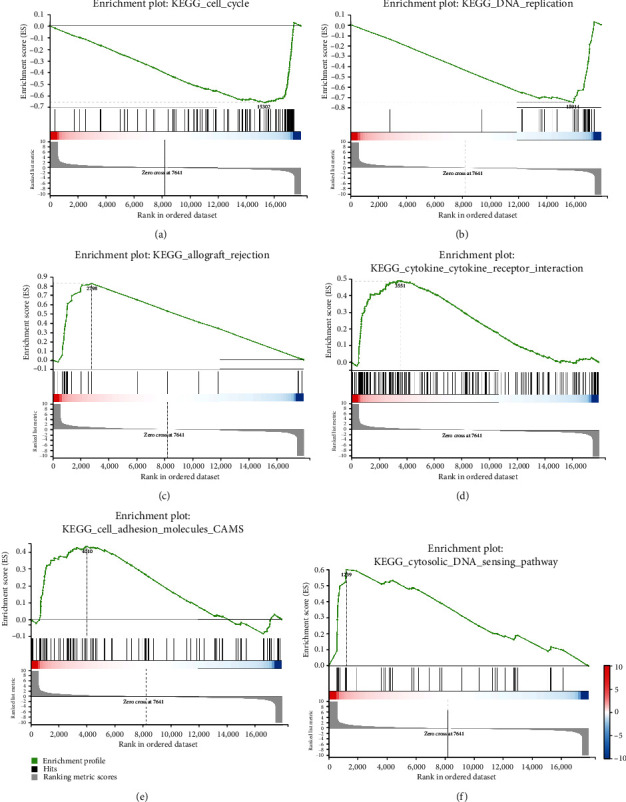
GSEA analysis of DEGs induced by palbociclib in NSCLC cells. (a) The genes upregulated in control cells were enriched in cell cycle pathway. (b) The genes upregulated in control cells were enriched in DNA replication-related pathway. The genes upregulated in palbociclib-treated cells were enriched in allograft rejection (c), cytokine-cytokine receptor interaction pathway (d), cell adhesion molecules (e), cytosolic DNA-sensing pathway (f), and so on.

Furthermore, we performed GSEA analysis. The results showed the genes upregulated in control cells were enriched in cell cycle (Figure 4(a) and Supplementary Table 4) and DNA replication-related pathways (Figure 4(b)), and the genes upregulated in palbociclib-treated cells were enriched in infection-/immune-related pathways (allograft rejection, autoimmune thyroid disease, graft-versus-host disease, and so on), cytokine-cytokine receptor interaction pathway, cell adhesion molecules, cytosolic DNA-sensing pathway, and so on (Figures 4(c)–4(f) and Supplementary Table 5).

**Figure 5:**
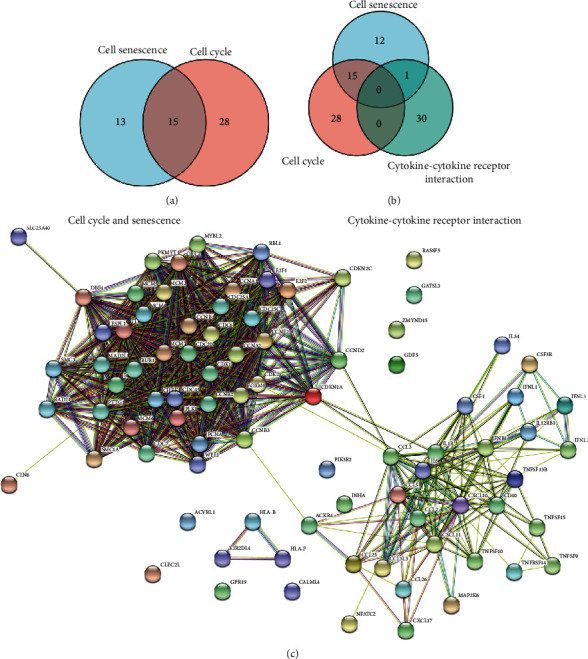
PPI analysis of DEGs induced by palbociclib in NSCLC cells. (a and b) The Venn diagram results showed the shared DEGs among cell cycle, cell senescence, and cytokine-cytokine receptor interaction pathway after treatment with 2 *μ*M palbociclib. (c) The interaction network about the genes enriched in cell cycle, cell senescence, and cytokine-cytokine receptor interaction pathways was built by STRING V11.

For the interaction of the pathways, we compared the genes between cell cycle and cell senescence (Figure 5(a)) and the genes among cell cycle, cell senescence, and cytokine-cytokine receptor interaction pathway (Figure 5(b)) and furtherly built the interaction network using the genes in the three pathways by STRING (version 11.5) [27]. The results showed that the genes related to cell cycle and cell senescence were enriched together and related to the genes in cytokine-cytokine receptor interaction pathway (Figure 5(c)). All the results indicated that CDK4/6 inhibitor could arrest cell cycle and induce cell senescence, resulting in inflammatory modules production which might enhance the invasion and migration abilities of NSCLC cells.

### 3.4. Blocking SASP-Related CCL5 Inhibits the Invasion Ability Induced by Palbociclib

**Figure 6:**
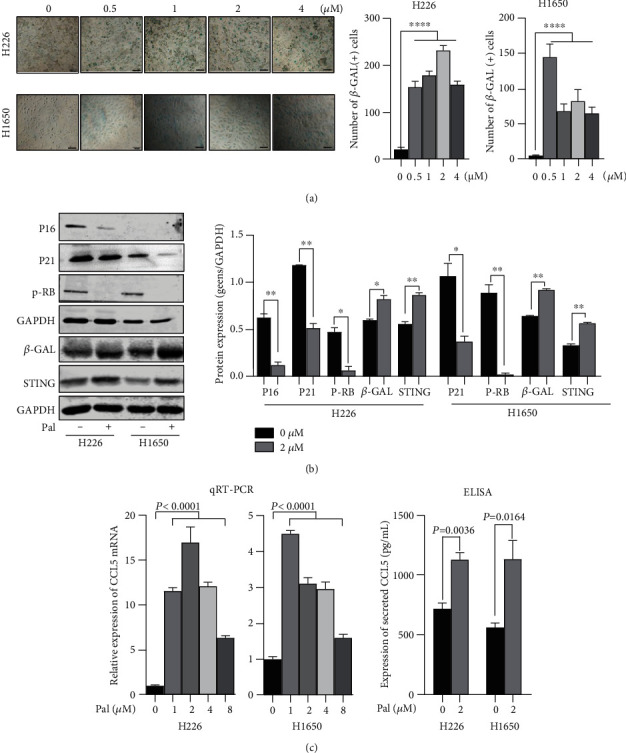
Palbociclib induces SASP-related CCL5 in NSCLC cells. (a) The *β*-galactosidase staining in NSCLC cells induced by palbociclib. Left: images of *β*-Gal-stained H1650 and H226 cells with/without palbociclib. Scale bars represent 100 *μ*m. Right: the bar charts display the calculated numbers of *β*-Gal positive cells. (b) Protein expression levels of the genes involved in cell senescence after treatment with 2 *μ*M palbociclib. Left: gel images of western blot for genes' expression, with GAPDH as a loading control. Right: the bar charts display the relative expression of target genes on protein level. P16, P21, p-RB, and corresponding GAPDH were tested in the same experiment. And *β*-Gal, STING, and corresponding GAPDH were tested in another experiment. Western blot result of every target protein cropped from the same gel in the same experiment and the proteins with similar size came from different gels in the same experiment. (c) CCL5 mRNA expression levels in NSCLC cells treated with palbociclib of gradient concentration (left) and secreted CCL5 in the supernatants of NSCLC cell with 2 *μ*M palbociclib (right). ∗*P* < 0.05, ∗∗*P* < 0.01, ∗∗∗*P* < 0.001, and ∗∗∗∗*P* < 0.0001.

SA-*β*-Gal activity is a critical marker of both senescence and aging. To validate the cell senescence induced by palbociclib, we performed *β*-galactosidase staining in the two cell lines. The results showed that treated with 2*μΜ* palbociclib, most H1650 and H226 cells were stained with blue and showed an upregulated *β*-Gal activity (Figure 6(a)). Western blot results also showed that palbociclib resulted in the downregulation of P16 and P21 and phosphorylated RB and the upregulation of *β*-Gal and STING (Figure 6(b)).

**Figure 7:**
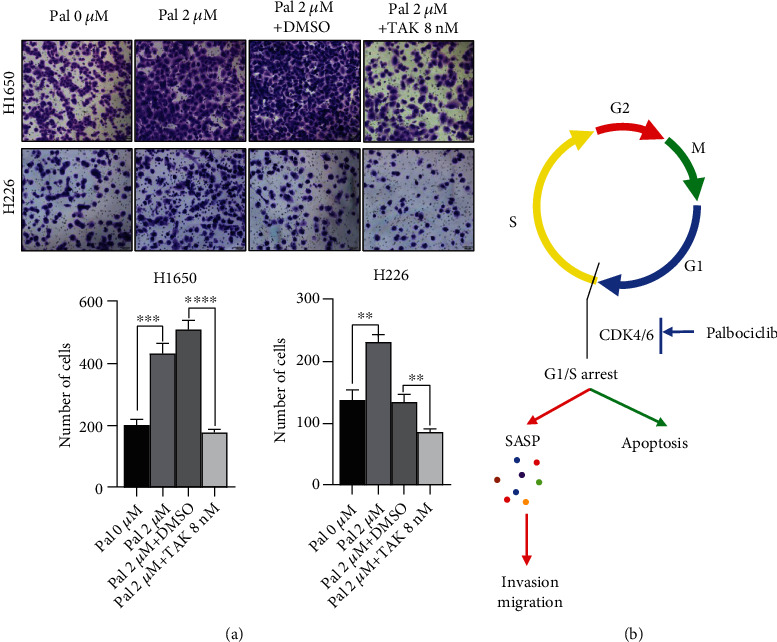
Blocking SASP-related CCL5 inhibits the migration ability induced by Palbociclib. (a) The effect of antagonists of CCR5 TAK-779 on the migration ability of NSCLC cells treated with 2 *μ*M Palbociclib. Upper: Images of migrated cell with Palbociclib or TAK-779, bar: 100 *μ*m. Lower: The results were analyzed using unpaired *t*-test. (b) Diagram showing how Palbociclib contributes to the migration and invasion of NSCLC via SASP-related CCL5.

Considering the relation between cytokine and cell migration/invasion, we screened the genes involved in cytokine-cytokine receptor interaction and found that CCL5 was upregulated significantly in the two cell lines after palbociclib treatment. qPCR assays showed that CCL5 mRNA was significantly increased after palbociclib treatment, and ELISA also showed that secreted CCL5 was significantly increased in the supernatant of palbociclib-treated cells (Figure 6(c)). Thus, CCL5 was selected as the candidate target gene. We treated the NSCLC cells with TAK-779 after palbociclib treatment and then performed transwell assays. The results showed that when CCL5 receptor was blocked, the migration ability of cancer cells induced by palbociclib was decreased significantly (Figure 7(a)). It indicated that the effect of palbociclib was partially dependent on the CCL5/CCR5 axis. In a conclusion, our results indicated that effective dose of palbociclib could arrest the cell cycle and induce apoptosis of cancer cells; however, palbociclib might induce cell senescence and enhance migration and invasion of cancer cells via SASP-related autocrine CCL5 (Figure 7(b)).

## 4. Discussion

As the first CDK4/6 inhibitor approved by FDA, palbociclib has been applied in clinical trials of many cancer types. However, the relationship between palbociclib and EMT was uncertain. Although Liu and Korc found that palbociclib enhanced cancer cells' invasiveness via inducing EMT in pancreatic cancer cells [10], most studies showed that palbociclib inhibited EMT process in various cancer types [8, 9, 28–30]. To solve this puzzle, we treated NSCLC cells with palbociclib and found that palbociclib inhibited growth and induces apoptosis of NSCLC cells; meanwhile, it enhanced the abilities of invasion and migration in NSCLC cells indeed. Our finding is partially consistent with Liu and Korc's result. However, we did not find solid evidence about EMT in our study. Although EMT is considered as a key process in cancer invasion and metastasis [31], some studies showed it is not required for cancer metastasis [32, 33]. Our results revealed that palbociclib-induced invasion and migration might be due to SASP rather than EMT.

Senescence is induced by different stressors with irreversible cell cycle arrest [34]. In the process, senescent cells can express a variety of extracellular bioactive molecules that include cytokines, chemokines, proteases, and growth factors, referred to as SASP [35, 36]. In the treatment of cancers, chemotherapy-drug therapy can induce cell senescence and SASP [37], which play a tumor-suppressive or tumor-promoting role [38]. It has been reported that conditioned media from senescent cancer cells trigger EMT-like malignant phenotype of cancer cells and promoted cancer metastasis [39]. SASP also induces a microenvironment that facilitates tumor progress and immunosuppression [40, 41]. Senescence and SASP also can be induced by CDK4/6 inhibitors in various cancers [42–44]. Guan et al. reported that prolonged CDK4/6 inhibitor induced normal fibroblasts to represent a strong SASP to promote melanoma growth and suppress antitumor immunity in vivo models [45]. Palbociclib-induced SASP was also reported to increase platelet activity which facilitates cancer migration and invasion [46]. Our transcriptome sequencing results showed after treatment with palbociclib, genes related to cell cycles were downregulated and genes related to chemokine signaling pathway were upregulated, indicating the NSCLC cells were induced to SASP by palbociclib.

Through comparing the DEGs, we found that chemokine signaling pathway might involve in SASP induced by palbociclib, and CCL5 was one potential mediator of the process. As an important member of the CC subfamily of chemokines, CCL5 was upregulated in various types of cancer [47]. Some studies have reported the relationship between CDK4/6 and CCL5. CDK4/6 inhibitor promoted the recruitment of tumor-infiltrating lymphocytes by inducing CCL5 secretion in melanoma [48] and in breast cancer [49]. CCL5 was also secreted by senescent cells [50, 51] and acted as one of SASP factors involved in cell proliferation, migration, and angiogenesis [17, 52]. Also, CCL5 was found to be increased, and chemokine/chemokine receptor signature was enriched after CDK4/6 inhibitor treatment in a phase II study in HR^+^/HER2^−^ breast cancer patients [53] and in a mouse model of mammary tumors [49], which are in agreement with our findings. Thus, SASP and SASP-related CCL5 might be potential promoters in palbociclib-induced invasion, and CCL5 in serum might be a potential marker to monitor the effect of CDK4/6 inhibitors. Considering the potential risk of palbociclib inducing SASP which promotes NSCLC malignant phenotypes, novel treatment strategy should be designed and developed as the concept of synthetic lethality [54–56]. Selectively targeting senescent cancer cells or SASP-related factors might act as a possible adjuvant therapeutic strategy for NSCLC patients with CDK4/6 inhibitor treatment.

## 5. Conclusions

Altogether, we found palbociclib blockaded cell cycle and enhanced invasion and migration capabilities in NSCLC cells via SASP-associated CCL5. Our results suggested that the combination of CDK4/6 inhibitor with CCL5 inhibitor or senotherapy could be considered to improve the curative effect and reduce the side effect of CDK4/6 inhibitors in the treatment of cancer.

## Data Availability

The sequencing data have been saved in the National Genomics Data Center, China, National Center for Bioinformation, accession number: HRA001631. https://ngdc.cncb.ac.cn/gsa-human/browse/HRA001631.

## Supplementary Materials

Supplementary 1 Supporting information 1. Supplementary Table 1: antibodies and primers used in the study.

Supplementary 2 Supporting information 2. Supplementary Figure 1: effect of palbociclib on NSCLC cells. (a) Effect of palbociclib on the apoptosis of NSCLC cells. Apoptosis assay was performed using Annexin V/PI staining kit (left). The results were analyzed using unpaired *t*-test (right). (b and c) Effect of the conditioned media from 2 *μ*M palbociclib-treated H226 and/or H1650 cells on the migration and invasion ability of NSCLC cells (left, bar: 100 *μ*m). The results were analyzed using unpaired *t*-test (right).

Supplementary 3 Supporting information 3. Supplementary Table 2: expression change of genes in 2 *μ*M palbociclib-treated H226 and H1650 cells.

Supplementary 4 Supporting information 4. Supplementary Table 3: KEGG enrichment analysis of DEGs between control and 2 *μ*M palbociclib-treated NSCLC cells.

Supplementary 5 Supporting information 5. Supplementary Table 4: GSEA analysis in control cells (MSigDB C2).

Supplementary 6 Supporting information 6. Supplementary Table 5: GSEA analysis in treated cells (MSigDB C2).
