# Supplementary material for: Palbociclib Enhances Migration and Invasion of Cancer Cells via Senescence-Associated Secretory Phenotype-Related CCL5 in Non-Small-Cell Lung Cancer
Source: J Oncol. 2022 Sep 27;2022:2260625. doi: 10.1155/2022/2260625 (PMC10175017; doi:10.1155/2022/2260625)
Supplement: Supplementary 1 — Supporting information 1. Supplementary Table 1: antibodies and primers used in the study. [file 2260625.f1.pdf]

Supplementary Table 1 Antibodies and primers used in the study

Antibody

Protein		Catalog number
p-Rb(Ser7[illegible]	CST	
P53	Proteintecl	80077-1-RR
P16	Proteintecl	10883-1-AP
β-gal	Proteintecl	66586-1-Ig
p21	Sigma	SAB5700189
ECAD	Proteintecl	20874-1-AP
NCAD	Proteintecl	22018-1-AP
VIM	Proteintecl	10366-1-AP
SNAI1	Proteintecl	13099-1-AP
GAPDH	Proteintecl	10494-1-AP

qPCR primer

Gene Syml	forward pr	Reverse primer
CCL5	CCAGCA[illegible]	CTCTGGGTTGGCACACACTT
GAPDH	GGAGCG[illegible]	GGCTGTTGTCATACTTCTCATGG
