# Supplementary figures and images for: Palbociclib Enhances Migration and Invasion of Cancer Cells via Senescence-Associated Secretory Phenotype-Related CCL5 in Non-Small-Cell Lung Cancer
Source: J Oncol. 2022 Sep 27;2022:2260625. doi: 10.1155/2022/2260625 (PMC10175017; doi:10.1155/2022/2260625)

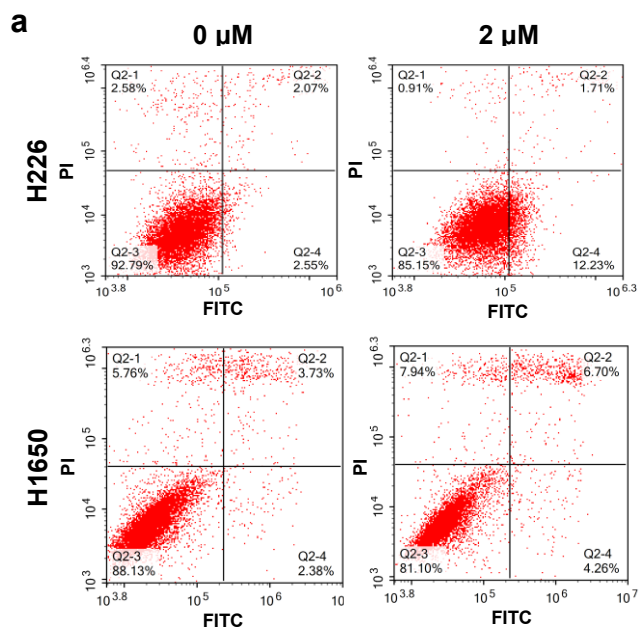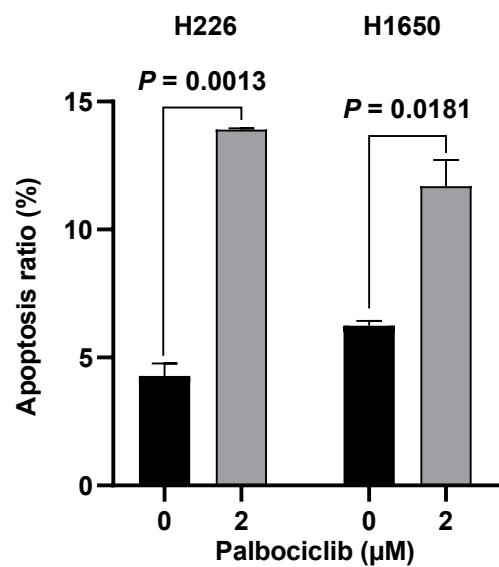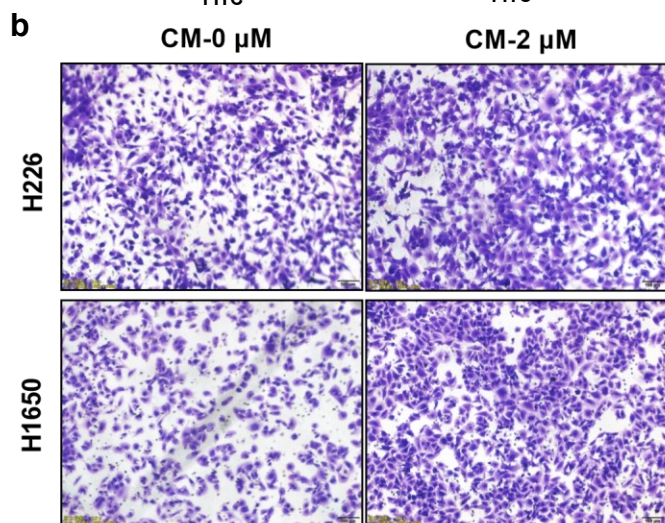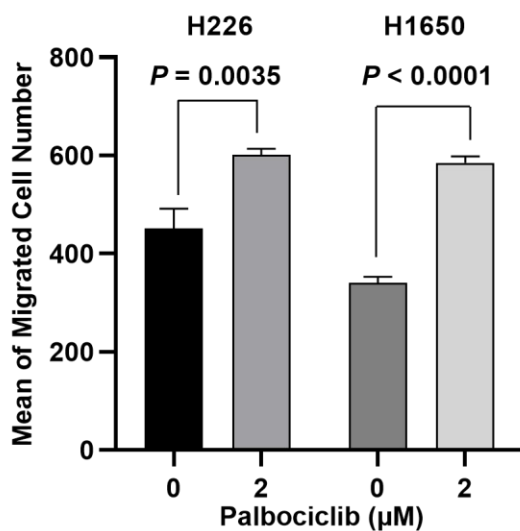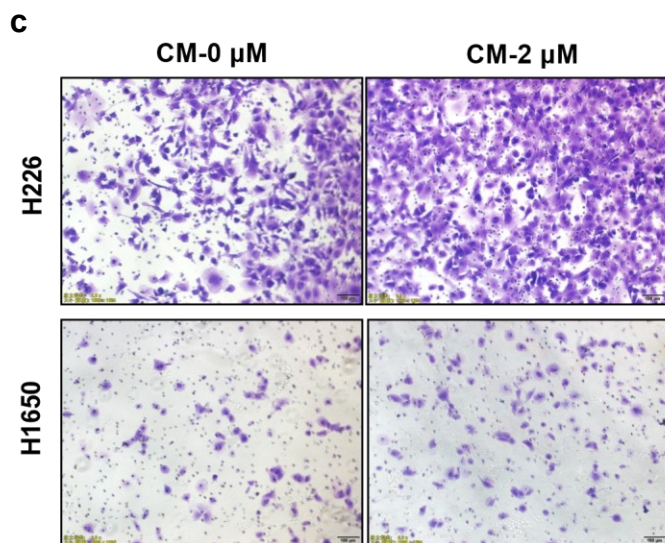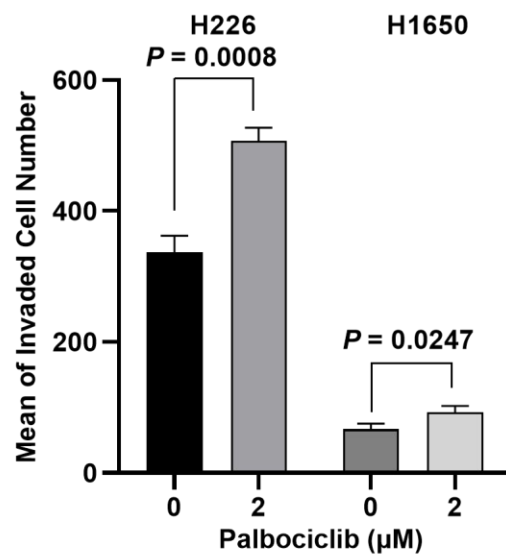

Supplement: Supplementary 2 — Supporting information 2. Supplementary Figure 1: effect of palbociclib on NSCLC cells. (a) Effect of palbociclib on the apoptosis of NSCLC cells. Apoptosis assay was performed using Annexin V/PI staining kit (left). The results were analyzed using unpaired t-test (right). (b and c) Effect of the conditioned media from 2 μM palbociclib-treated H226 and/or H1650 cells on the migration and invasion ability of NSCLC cells (left, bar: 100 μm). The results were analyzed using unpaired t-test (right). [file 2260625.f2.pdf]
