# Supplementary material for: Palbociclib Enhances Migration and Invasion of Cancer Cells via Senescence-Associated Secretory Phenotype-Related CCL5 in Non-Small-Cell Lung Cancer
Source: J Oncol. 2022 Sep 27;2022:2260625. doi: 10.1155/2022/2260625 (PMC10175017; doi:10.1155/2022/2260625)
Supplement: Supplementary 3 — Supporting information 3. Supplementary Table 2: expression change of genes in 2 μM palbociclib-treated H226 and H1650 cells. [file 2260625.f3.pdf]

**Supplementary Table 2 Expression change of Genes in 2 μM Palbociclib-treated H226 and H1650 cells.**

| Gene ID | Gene Symbol | log2 (H16502umol | FDR (H16502umol | log2 (H2262umol / | FDR (H2262ur | Pvalue(H165 | Pvalue(H2262umc | log2 (LUSCtreat / L | Pvalue | Qvalue |
|---|---|---|---|---|---|---|---|---|---|---|
| 100008586 | 'GAGE12F' | 6.741466986 | 7.36E-05 | -6.754887502 | 8.88E-05 | 3.13E-05 | 3.51E-05 | -0.013898224 | 0.978611649 | 0.999803685 |
| 100129083 | 'LOC100129083' | -2.46712601 | 6.18E-07 | -2.557995453 | 1.77E-04 | 2.23E-07 | 7.21E-05 | -2.541403063 | 1.89E-11 | 5.39E-11 |
| 10024 | 'TROAP' | -2.811401755 | 0 | -2.472030627 | 0 | 0 | 0 | -2.545777695 | 0 | 0 |
| 100462977 | 'MTRNR2L1' | 1.598937841 | 0 | 1.045244295 | 7.49E-302 | 0 | 9.15E-304 | 1.401944344 | 0 | 0 |
| 10051 | 'SMC4' | -1.481921624 | 3.88E-201 | -1.855989697 | 2.38E-187 | 1.04E-202 | 5.02E-189 | -1.642143345 | 0 | 0 |
| 100526740 | 'ATP5MF-PTCD1' | 4 | 9.10E-08 | 3.321928095 | 3.47E-05 | 3.10E-08 | 1.32E-05 | 6.419369452 | 1.43E-11 | 4.11E-11 |
| 100528030 | 'POC1B-GALNT4 | -5.930737338 | 1.45E-28 | -2.44625623 | 2.92E-23 | 2.17E-29 | 4.52E-24 | -3.048073313 | 5.51E-47 | 3.88E-46 |
| 100533183 | 'ZNF664-RFLNA' | -4.321928095 | 1.37E-04 | -2.602036014 | 7.41E-10 | 5.96E-05 | 2.02E-10 | -2.971805449 | 6.23E-14 | 1.97E-13 |
| 10112 | 'KIF20A' | -3.788586286 | 0 | -2.49259275 | 0 | 0 | 0 | -2.909155855 | 0 | 0 |
| 1017 | 'CDK2' | -1.370589528 | 5.54E-218 | -1.007320407 | 5.34E-187 | 1.36E-219 | 1.13E-188 | -1.152689987 | 0 | 0 |
| 101927601 | 'GOLGA6L19' | 4.584962501 | 1.59E-09 | -3.807354922 | 1.24E-05 | 4.89E-10 | 4.50E-06 | 0.741473679 | 0.072842216 | 0.103657252 |
| 10202 | 'DHRS2' | -2.033947332 | 7.52E-15 | -2.578076115 | 3.43E-47 | 1.80E-15 | 2.96E-48 | -2.485639481 | 9.40E-62 | 8.29E-61 |
| 10212 | 'DDX39A' | -1.578416626 | 0 | -1.489819531 | 0 | 0 | 0 | -1.521595958 | 0 | 0 |
| 10232 | 'MSLN' | 2.207712711 | 0 | 2.2410081 | 8.51E-05 | 0 | 3.35E-05 | 2.215752551 | 0 | 0 |
| 1026 | 'CDKN1A' | -1.078724112 | 7.26E-147 | -1.345222055 | 2.02E-235 | 2.67E-148 | 3.36E-237 | -1.220703807 | 0 | 0 |
| 102724200 | 'LOC102724200' | 1.523561956 | 2.38E-10 | 4.169925001 | 3.47E-05 | 7.03E-11 | 1.32E-05 | 1.703039893 | 4.20E-14 | 1.34E-13 |
| 102724594 | 'U2AF1L5' | -3.062284278 | 1.74E-06 | -6.3594028 | 7.15E-112 | 6.50E-07 | 2.70E-113 | -4.803058482 | 2.49E-107 | 3.40E-106 |
| 10317 | 'B3GALT5' | -1.638901308 | 1.97E-08 | -2.807354922 | 2.54E-04 | 6.46E-09 | 1.05E-04 | -1.886588494 | 4.60E-12 | 1.35E-11 |
| 1033 | 'CDKN3' | -2.491275798 | 1.65E-225 | -2.167888016 | 9.88E-158 | 3.90E-227 | 2.55E-159 | -2.351905955 | 0 | 0 |
| 10382 | 'TUBB4A' | 2.162938571 | 7.74E-18 | 1.811097324 | 8.70E-102 | 1.64E-18 | 3.65E-103 | 1.83339915 | 5.78E-118 | 8.58E-117 |
| 10403 | 'NDC80' | -2.790772038 | 2.26E-170 | -1.786090105 | 2.60E-105 | 7.08E-172 | 1.05E-106 | -2.195542684 | 1.75E-266 | 5.78E-265 |
| 10516 | 'FBLN5' | 1.111031312 | 2.90E-08 | 1.448900951 | 3.49E-56 | 9.59E-09 | 2.59E-57 | 1.371854866 | 4.12E-63 | 3.70E-62 |
| 1058 | 'CENPA' | -2.200975747 | 2.66E-125 | -1.954326665 | 7.08E-113 | 1.14E-126 | 2.65E-114 | -2.083193872 | 4.70E-238 | 1.38E-236 |
| 10608 | 'MXD4' | 1.124771615 | 1.30E-234 | 1.497302208 | 0 | 2.96E-236 | 0 | 1.370493578 | 0 | 0 |
| 10615 | 'SPAG5' | -2.326834034 | 0 | -1.464134583 | 5.90E-223 | 0 | 1.05E-224 | -1.907420818 | 0 | 0 |
| 1062 | 'CENPE' | -1.665335917 | 2.32E-42 | -1.725825037 | 5.36E-29 | 2.57E-43 | 7.07E-30 | -1.69358556 | 3.27E-71 | 3.23E-70 |
| 1063 | 'CENPF' | -2.422761315 | 0 | -1.712970113 | 1.38E-85 | 0 | 7.06E-87 | -2.185029585 | 0 | 0 |
| 10635 | 'RAD51AP1' | -2.019590728 | 7.81E-72 | -1.365309441 | 2.55E-39 | 5.35E-73 | 2.58E-40 | -1.673309135 | 3.41E-108 | 4.69E-107 |
| 10690 | 'FUT9' | -2.321928095 | 9.37E-06 | -1.428843299 | 7.00E-10 | 3.70E-06 | 1.91E-10 | -1.611877393 | 7.82E-14 | 2.47E-13 |
| 10721 | 'POLQ' | -1.671616883 | 3.13E-42 | -1.133855747 | 3.01E-18 | 3.47E-43 | 5.56E-19 | -1.460602463 | 6.44E-59 | 5.46E-58 |
| 107303344 | 'SETDB2-PHF11' | -5.882643049 | 2.45E-19 | -3.169925001 | 3.38E-04 | 4.90E-20 | 1.42E-04 | -5.33648475 | 2.01E-21 | 8.19E-21 |
| 10733 | 'PLK4' | -1.901281949 | 4.41E-79 | -1.807354922 | 6.14E-42 | 2.78E-80 | 5.87E-43 | -1.859186166 | 9.17E-121 | 1.39E-119 |
| 10785 | 'WDR4' | -1.426181499 | 2.18E-38 | -1.453276304 | 7.37E-32 | 2.63E-39 | 9.00E-33 | -1.410092658 | 2.72E-70 | 2.66E-69 |
| 107986354 | 'LOC107986354' | 1.657112286 | 1.75E-04 | -3.700439718 | 3.29E-04 | 7.69E-05 | 1.38E-04 | 0.680539603 | 0.052803601 | 0.076819669 |
| 10874 | 'NMU' | -3.067976794 | 4.46E-18 | -1.190778317 | 2.28E-04 | 9.37E-19 | 9.38E-05 | -2.024447405 | 1.57E-19 | 6.06E-19 |
| 10926 | 'DBF4' | -1.288676289 | 4.21E-51 | -1.426968451 | 2.67E-58 | 3.94E-52 | 1.91E-59 | -1.3617458 | 1.48E-109 | 2.05E-108 |
| 10964 | 'IFI44L' | 2.06871275 | 9.11E-84 | 6.06608919 | 2.20E-17 | 5.43E-85 | 4.18E-18 | 2.1999205 | 3.96E-98 | 5.04E-97 |
| 11004 | 'KIF2C' | -2.810396362 | 0 | -1.308846504 | 1.720216264E | 0 | 1.9877417E-317 | -1.854399833 | 0 | 0 |
| 11013 | 'TMSB15A' | -2.873276315 | 8.15E-12 | -2.807354922 | 6.98E-09 | 2.23E-12 | 2.01E-09 | -2.872325036 | 1.70E-20 | 6.73E-20 |
| 11045 | 'UPK1A' | 1.637429921 | 1.55E-06 | 2.115477217 | 5.80E-07 | 5.77E-07 | 1.89E-07 | 1.823549502 | 4.50E-13 | 1.38E-12 |
| 11064 | 'CNTRL' | -2.014950341 | 9.21E-40 | -1.707819249 | 2.79E-10 | 1.08E-40 | 7.40E-11 | -1.977298262 | 1.62E-49 | 1.20E-48 |
| 11065 | 'UBE2C' | -1.919934977 | 0 | -1.867919423 | 0 | 0 | 0 | -1.901340788 | 0 | 0 |
| 11067 | 'DEPP1' | 1.92087135 | 1.20E-20 | 1.274307433 | 2.30E-57 | 2.28E-21 | 1.67E-58 | 1.342201827 | 2.67E-75 | 2.75E-74 |
| 1111 | 'CHEK1' | -1.337902327 | 1.16E-127 | -1.085391491 | 9.29E-58 | 4.84E-129 | 6.71E-59 | -1.197099777 | 5.00E-183 | 1.11E-181 |
| 11130 | 'ZWINT' | -2.205204489 | 5.13E-252 | -1.724876704 | 4.87E-300 | 1.08E-253 | 6.03E-302 | -1.89249573 | 0 | 0 |
| 11144 | 'DMC1' | -3.210566986 | 7.55E-35 | -2.847996907 | 4.22E-18 | 9.71E-36 | 7.85E-19 | -3.01863336 | 2.96E-52 | 2.28E-51 |
| 11169 | 'WDHD1' | -1.841496178 | 2.08E-93 | -1.019899557 | 1.79E-11 | 1.13E-94 | 4.45E-12 | -1.600619346 | 1.01E-100 | 1.30E-99 |
| 1122 | 'CHML' | -1.483815777 | 3.03E-27 | -1.126532406 | 1.37E-13 | 4.70E-28 | 3.06E-14 | -1.324765446 | 4.77E-40 | 2.98E-39 |
| 112267923 | '112267923' | 4.392317423 | 1.94E-05 | -4.392317423 | 2.39E-05 | 7.85E-06 | 8.92E-06 | 0.005365966 | 0.991226729 | 1 |
| 11248 | 'NXPH3' | 1.584962501 | 8.08E-08 | 2 | 3.62E-06 | 2.75E-08 | 1.25E-06 | 1.700633514 | 1.26E-13 | 3.94E-13 |
| 11249 | 'NXPH2' | -1.121990524 | 1.98E-13 | -2.118644496 | 1.44E-13 | 5.04E-14 | 3.21E-14 | -1.339624458 | 1.04E-24 | 4.66E-24 |
| 11270 | 'NRM' | -1.430843595 | 3.02E-47 | -1.015905883 | 2.52E-107 | 3.01E-48 | 9.94E-109 | -1.124570174 | 2.27E-154 | 4.35E-153 |
| 11340 | 'EXOSC8' | -1.17663097 | 1.56E-34 | -1.458522643 | 1.89E-61 | 2.02E-35 | 1.30E-62 | -1.331914178 | 1.03E-95 | 1.28E-94 |
| 113451 | 'AZIN2' | 1.245207409 | 9.79E-35 | 1.001937804 | 4.24E-18 | 1.26E-35 | 7.88E-19 | 1.181973073 | 1.18E-52 | 9.12E-52 |
| 113655 | 'MFSD3' | 1.3037249 | 1.33E-85 | 1.027928744 | 1.47E-37 | 7.70E-87 | 1.55E-38 | 1.175919682 | 1.49E-122 | 2.29E-121 |
| 113791 | 'PIK3IP1' | 1.231005552 | 9.14E-16 | 2.353887836 | 5.63E-59 | 2.11E-16 | 3.98E-60 | 1.815708749 | 1.36E-69 | 1.32E-68 |
| 1138 | 'CHRNA5' | -1.799249872 | 8.22E-36 | -1.078002512 | 3.61E-11 | 1.04E-36 | 9.14E-12 | -1.480668339 | 1.54E-44 | 1.04E-43 |
| 114822 | 'RHPN1' | 1.387516437 | 8.03E-18 | 1.091814866 | 8.19E-70 | 1.71E-18 | 4.99E-71 | 1.099936019 | 1.92E-85 | 2.19E-84 |
| 114898 | 'C1QTNF2' | -1.115477217 | 2.34E-04 | -1.439006147 | 1.78E-13 | 1.04E-04 | 4.01E-14 | -1.377133654 | 1.33E-17 | 4.80E-17 |
| 115703 | 'ARHGAP33' | -1.511237007 | 1.18E-76 | -1.467005512 | 2.25E-207 | 7.66E-78 | 4.36E-209 | -1.511333823 | 1.12E-287 | 4.05E-286 |
| 121268 | 'RHEBL1' | 1.084888898 | 2.94E-13 | -1.562152618 | 2.53E-07 | 7.54E-14 | 8.05E-08 | 0.532152536 | 2.49E-05 | 5.11E-05 |
| 122622 | 'ADSSL1' | 1.153656612 | 2.03E-12 | 1.253122166 | 3.54E-160 | 5.41E-13 | 9.02E-162 | 1.212344676 | 2.18E-169 | 4.52E-168 |
| 122769 | 'LRR1' | -1.216905235 | 1.22E-29 | -1.287143182 | 2.49E-18 | 1.78E-30 | 4.59E-19 | -1.142006357 | 1.58E-47 | 1.12E-46 |
| 123099 | 'DEGS2' | 2.500265336 | 1.20E-12 | 2.021061616 | 1.45E-09 | 3.16E-13 | 4.04E-10 | 2.247357211 | 7.82E-22 | 3.23E-21 |
| 124222 | 'PAQR4' | -1.330127365 | 4.01E-88 | -1.586225806 | 3.69E-124 | 2.29E-89 | 1.22E-125 | -1.45372616 | 1.54E-212 | 4.01E-211 |
| 124936 | 'CYB5D2' | 1.05828985 | 8.27E-56 | 1.017135912 | 1.54E-21 | 7.11E-57 | 2.50E-22 | 1.00960855 | 1.48E-78 | 1.57E-77 |
| 126147 | 'NTN5' | 1.386779239 | 1.93E-17 | 3.551795637 | 8.32E-15 | 4.15E-18 | 1.76E-15 | 1.642586229 | 3.89E-29 | 1.93E-28 |
| 127602 | 'DNAH14' | -1.165662975 | 2.65E-07 | -1.951978485 | 8.85E-10 | 9.28E-08 | 2.43E-10 | -1.034325902 | 9.19E-16 | 3.11E-15 |
| 128239 | 'IQGAP3' | -2.768249524 | 0 | -1.923218766 | 1.35E-232 | 0 | 2.28E-234 | -2.273515888 | 0 | 0 |
| 128434 | 'VSTM2L' | 1.674599713 | 4.98E-195 | 1.925999419 | 2.96E-12 | 1.38E-196 | 7.08E-13 | 1.699341545 | 7.12E-211 | 1.83E-209 |
| 129080 | 'EMID1' | 1.094327383 | 9.76E-11 | 1.666756592 | 1.24E-06 | 2.83E-11 | 4.13E-07 | 1.190890259 | 8.69E-17 | 3.04E-16 |
| 1291 | 'COL6A1' | 1.138139668 | 0 | 1.02600072 | 1.47E-229 | 0 | 2.51E-231 | 1.12042604 | 0 | 0 |
| 129303 | 'TMEM150A' | 1.175168571 | 4.21E-48 | 1.883259366 | 2.28E-78 | 4.14E-49 | 1.26E-79 | 1.399946349 | 1.10E-120 | 1.68E-119 |
| 130733 | 'TMEM178A' | 3.227068909 | 4.20E-10 | 1.823122238 | 3.12E-06 | 1.26E-10 | 1.07E-06 | 2.187023752 | 5.48E-15 | 1.81E-14 |
| 132320 | 'SCLT1' | -1.505374236 | 2.95E-29 | -1.603920658 | 2.34E-17 | 4.32E-30 | 4.45E-18 | -1.419518638 | 2.78E-46 | 1.93E-45 |
| 134265 | 'AFAP1L1' | -1.165766177 | 5.59E-17 | -1.478047297 | 2.25E-12 | 1.23E-17 | 5.35E-13 | -0.940172887 | 1.79E-28 | 8.77E-28 |

| | | | | | | | | | | |
|---|---|---|---|---|---|---|---|---|---|---|
| 139886 | 'SPIN4' | -1.327804661 | 5.44E-36 | -1.538095896 | 3.59E-57 | 6.84E-37 | 2.61E-58 | -1.44521267 | 2.94E-93 | 3.58E-92 |
| 140606 | 'SELENOM' | 1.422324396 | 0 | 1.184473197 | 5.37E-91 | 0 | 2.55E-92 | 1.381684072 | 0 | 0 |
| 1417 | 'CRYBB3' | 3.94016675 | 4.85E-129 | -1.201633861 | 4.37E-14 | 2.01E-130 | 9.54E-15 | 1.216941073 | 2.54E-38 | 1.54E-37 |
| 143888 | 'KDELC2' | -1.024158869 | 4.37E-257 | -1.418889825 | 3.83E-82 | 8.85E-259 | 2.02E-83 | -1.068599146 | 0 | 0 |
| 144110 | 'TMEM86A' | 1.017921908 | 9.55E-08 | 2.285402219 | 4.77E-32 | 3.26E-08 | 5.80E-33 | 1.633078642 | 1.29E-35 | 7.43E-35 |
| 144455 | 'E2F7' | -1.599289282 | 7.05E-127 | -1.989216324 | 1.85E-167 | 2.96E-128 | 4.46E-169 | -1.757049059 | 3.24E-291 | 1.19E-289 |
| 145773 | 'FAM81A' | -1.286881148 | 1.97E-04 | -1.893084796 | 3.88E-22 | 8.71E-05 | 6.20E-23 | -1.743925572 | 4.89E-26 | 2.25E-25 |
| 145781 | 'GCOM1' | 5.392317423 | 3.90E-20 | 3.700439718 | 2.34E-06 | 7.53E-21 | 7.94E-07 | 7.461034402 | 5.25E-21 | 2.11E-20 |
| 145864 | 'HAPLN3' | 2.075399778 | 2.66E-65 | 1.020177882 | 4.79E-10 | 1.99E-66 | 1.29E-10 | 1.744887104 | 6.64E-72 | 6.60E-71 |
| 146909 | 'KIF18B' | -2.354133708 | 0 | -2.332025319 | 0 | 0 | 0 | -2.324277376 | 0 | 0 |
| 147699 | 'PPM1N' | 1.06153841 | 3.40E-12 | 1.082826431 | 4.42E-20 | 9.14E-13 | 7.59E-21 | 1.03315799 | 9.68E-32 | 5.10E-31 |
| 148170 | 'CDC42EP5' | 1.707377709 | 2.16E-166 | 3.216317907 | 2.41E-07 | 6.92E-168 | 7.67E-08 | 1.728156018 | 4.73E-176 | 1.01E-174 |
| 150468 | 'CKAP2L' | -2.475938607 | 5.44E-166 | -1.977912379 | 3.55E-99 | 1.75E-167 | 1.55E-100 | -2.20231984 | 3.36E-262 | 1.10E-260 |
| 151246 | 'SGO2' | -1.948036578 | 4.46E-53 | -1.471305719 | 5.82E-17 | 4.01E-54 | 1.12E-17 | -1.757101377 | 6.23E-69 | 5.98E-68 |
| 151556 | 'GPR155' | 1.444784843 | 3.97E-09 | 1.714245518 | 6.13E-17 | 1.25E-09 | 1.18E-17 | 1.581605577 | 1.08E-25 | 4.94E-25 |
| 151648 | 'SGO1' | -1.817408587 | 9.21E-72 | -1.205258075 | 1.77E-20 | 6.32E-73 | 2.99E-21 | -1.550976929 | 6.63E-89 | 7.77E-88 |
| 157313 | 'CDCA2' | -1.856922136 | 2.45E-95 | -1.370022383 | 8.66E-50 | 1.31E-96 | 7.19E-51 | -1.605826142 | 2.29E-142 | 4.06E-141 |
| 157570 | 'ESCO2' | -3.44531051 | 1.62E-57 | -2.351675438 | 8.11E-32 | 1.35E-58 | 9.92E-33 | -2.843012337 | 3.94E-87 | 4.54E-86 |
| 158067 | 'AK8' | 1.315904307 | 2.46E-06 | 1.508223057 | 1.42E-13 | 9.30E-07 | 3.17E-14 | 1.268892771 | 2.54E-19 | 9.69E-19 |
| 161145 | 'TMEM229B' | 1.561503528 | 8.95E-17 | 1.056583528 | 7.73E-31 | 1.98E-17 | 9.77E-32 | 1.146295683 | 1.13E-45 | 7.77E-45 |
| 163175 | 'LGI4' | 1.301169535 | 5.42E-05 | 1.247927513 | 2.31E-07 | 2.27E-05 | 7.34E-08 | 1.166822499 | 7.55E-12 | 2.20E-11 |
| 168544 | 'ZNF467' | 1.673318375 | 6.60E-252 | 1.167798902 | 1.83E-153 | 1.39E-253 | 4.87E-155 | 1.306309085 | 0 | 0 |
| 170961 | 'ANKRD24' | 1.010124217 | 1.13E-05 | 1.127111918 | 1.21E-13 | 4.50E-06 | 2.70E-14 | 1.052138189 | 1.12E-18 | 4.18E-18 |
| 1719 | 'DHFR' | -2.175171171 | 0 | -1.852046412 | 0 | 0 | 0 | -1.991695827 | 0 | 0 |
| 1786 | 'DNMT1' | -1.392760579 | 0 | -1.110537279 | 0 | 0 | 0 | -1.234013927 | 0 | 0 |
| 1843 | 'DUSP1' | 1.512302247 | 0 | 1.362208999 | 2.15E-139 | 0 | 6.38E-141 | 1.470401128 | 0 | 0 |
| 1869 | 'E2F1' | -1.352129984 | 0 | -1.463224957 | 4.12E-173 | 0 | 9.57E-175 | -1.379023003 | 0 | 0 |
| 1870 | 'E2F2' | -3.85655892 | 2.81E-135 | -1.889917517 | 8.61E-105 | 1.10E-136 | 3.53E-106 | -2.288991779 | 1.26E-220 | 3.39E-219 |
| 1894 | 'ECT2' | -1.132862907 | 2.78E-92 | -1.294467872 | 2.25E-92 | 1.53E-93 | 1.05E-93 | -1.20892687 | 5.20E-184 | 1.17E-182 |
| 195828 | 'ZNF367' | -2.768826693 | 6.11E-120 | -2.116767066 | 6.70E-140 | 2.70E-121 | 1.97E-141 | -2.365445481 | 1.05E-257 | 3.34E-256 |
| 197257 | 'LDHD' | 2.048094288 | 1.37E-15 | 2.050626073 | 3.27E-50 | 3.19E-16 | 2.68E-51 | 2.020033168 | 2.81E-65 | 2.57E-64 |
| 201191 | 'SAMD14' | 1.074989896 | 1.80E-27 | 1.216811389 | 2.18E-12 | 2.78E-28 | 5.19E-13 | 1.133822967 | 3.70E-40 | 2.31E-39 |
| 201229 | 'LYRM9' | 1.474602053 | 7.19E-16 | 2.073248982 | 8.92E-19 | 1.65E-16 | 1.61E-19 | 1.642586229 | 3.97E-34 | 2.22E-33 |
| 2013 | 'EMP2' | -1.241230036 | 8.14E-97 | -1.063502942 | 1.61E-27 | 4.31E-98 | 2.21E-28 | -1.186153391 | 8.51E-123 | 1.31E-121 |
| 203190 | 'LGI3' | 1.567040593 | 9.68E-07 | 3.807354922 | 2.61E-04 | 3.56E-07 | 1.08E-04 | 1.801731392 | 8.15E-10 | 2.16E-09 |
| 2146 | 'EZH2' | -2.137717114 | 1.39E-129 | -1.428550514 | 6.65E-97 | 5.69E-131 | 2.95E-98 | -1.690809128 | 2.42E-219 | 6.47E-218 |
| 2175 | 'FANCA' | -1.562313762 | 3.46E-131 | -1.396762217 | 2.22E-263 | 1.40E-132 | 3.10E-265 | -1.453503829 | 0 | 0 |
| 218 | 'ALDH3A1' | 1.214594051 | 8.46E-04 | 1.033947332 | 3.17E-05 | 3.95E-04 | 1.20E-05 | 1.062785433 | 1.84E-08 | 4.57E-08 |
| 2199 | 'FBLN2' | 1.000784998 | 9.57E-253 | 1.365195549 | 1.81E-83 | 1.99E-254 | 9.38E-85 | 1.063833523 | 0 | 0 |
| 220134 | 'SKA1' | -2.328735566 | 2.61E-161 | -1.858230964 | 1.37E-116 | 8.59E-163 | 4.86E-118 | -2.080428834 | 2.03E-275 | 6.95E-274 |
| 221150 | 'SKA3' | -3.004236993 | 2.23E-98 | -2.448967283 | 5.14E-149 | 1.16E-99 | 1.42E-150 | -2.625207826 | 2.36E-246 | 7.22E-245 |
| 2217 | 'FCGRT' | 1.123988717 | 5.26E-158 | 1.174171588 | 1.30E-98 | 1.76E-159 | 5.72E-100 | 1.144764595 | 4.83E-259 | 1.56E-257 |
| 222865 | 'TMEM130' | 1.737581999 | 1.05E-179 | 2.584962501 | 4.34E-06 | 3.15E-181 | 1.52E-06 | 1.758434065 | 1.54E-188 | 3.53E-187 |
| 2237 | 'FEN1' | -1.461252991 | 5.57E-241 | -1.260312825 | 5.69E-141 | 1.22E-242 | 1.66E-142 | -1.375629114 | 0 | 0 |
| 2246 | 'FGF1' | 2.499232627 | 5.45E-23 | -1.12882998 | 6.17E-11 | 9.54E-24 | 1.58E-11 | 0.072439725 | 0.570402205 | 0.637809609 |
| 22801 | 'ITGA11' | 1.184968882 | 6.73E-20 | 1.479167837 | 1.75E-19 | 1.32E-20 | 3.09E-20 | 1.282801483 | 3.61E-39 | 2.23E-38 |
| 22974 | 'TPX2' | -2.105168919 | 0 | -1.139257865 | 0 | 0 | 0 | -1.5441128 | 0 | 0 |
| 22995 | 'CEP152' | -1.287162677 | 2.79E-22 | -2.128104826 | 6.04E-27 | 5.00E-23 | 8.44E-28 | -1.64390715 | 9.31E-48 | 6.65E-47 |
| 2305 | 'FOXM1' | -2.166785294 | 0 | -1.790576964 | 0 | 0 | 0 | -1.908193754 | 0 | 0 |
| 23178 | 'PASK' | -1.473515965 | 5.73E-32 | -2.01227833 | 4.29E-62 | 7.86E-33 | 2.90E-63 | -1.710676624 | 9.01E-93 | 1.09E-91 |
| 23234 | 'DNAJC9' | -1.174164484 | 5.53E-128 | -1.025822385 | 5.91E-106 | 2.30E-129 | 2.38E-107 | -1.102568242 | 3.66E-234 | 1.05E-232 |
| 23397 | 'NCAPH' | -2.925893068 | 0 | -1.433765954 | 2.29E-141 | 0 | 6.67E-143 | -2.053000019 | 0 | 0 |
| 2353 | 'FOS' | 1.007876426 | 3.57E-39 | 2.045081799 | 0 | 4.24E-40 | 0 | 1.847155957 | 0 | 0 |
| 23586 | 'DDX58' | 1.541770127 | 4.87E-248 | 1.092869575 | 4.16E-25 | 1.04E-249 | 6.14E-26 | 1.475929764 | 2.54E-274 | 8.68E-273 |
| 23646 | 'PLD3' | 1.487555771 | 0 | 1.634241212 | 0 | 0 | 0 | 1.573499112 | 0 | 0 |
| 24137 | 'KIF4A' | -2.773124172 | 0 | -1.24346572 | 7.92E-257 | 0 | 1.17E-258 | -1.668190477 | 0 | 0 |
| 2491 | 'CENPI' | -1.863081234 | 7.92E-85 | -1.501397784 | 4.19E-52 | 4.65E-86 | 3.32E-53 | -1.674242654 | 1.04E-136 | 1.77E-135 |
| 2521 | 'FUS' | -1.507312352 | 0 | -1.250438547 | 0 | 0 | 0 | -1.342598464 | 0 | 0 |
| 2537 | 'IFI6' | 2.066669909 | 0 | 1.918933509 | 1.04E-161 | 0 | 2.59E-163 | 2.03388054 | 0 | 0 |
| 253714 | 'MMS22L' | -1.714495313 | 2.80E-63 | -1.091147888 | 5.41E-50 | 2.16E-64 | 4.47E-51 | -1.309739044 | 1.71E-111 | 2.42E-110 |
| 2548 | 'GAA' | 1.166746241 | 0 | 1.19246202 | 6.24E-293 | 0 | 7.88E-295 | 1.179407178 | 0 | 0 |
| 255809 | 'C19orf38' | 2.234169589 | 5.54E-11 | 2.183797176 | 1.55E-09 | 1.58E-11 | 4.30E-10 | 2.099955226 | 2.65E-20 | 1.04E-19 |
| 256126 | 'SYCE2' | -1.4246662 | 5.86E-24 | -1.584962501 | 6.76E-14 | 9.97E-25 | 1.49E-14 | -1.480668339 | 2.09E-37 | 1.25E-36 |
| 256472 | 'TMEM151A' | 1.023478062 | 1.96E-70 | 1.129767875 | 3.96E-07 | 1.37E-71 | 1.28E-07 | 1.040034719 | 1.39E-79 | 1.49E-78 |
| 25759 | 'SHC2' | 1.918749679 | 5.69E-29 | 1.473286264 | 4.20E-25 | 8.44E-30 | 6.19E-26 | 1.643489607 | 3.92E-53 | 3.05E-52 |
| 25789 | 'TMEM59L' | 1.340637224 | 1.19E-32 | 2.725825037 | 7.58E-19 | 1.61E-33 | 1.37E-19 | 1.480947839 | 1.18E-48 | 8.55E-48 |
| 259173 | 'ALS2CL' | 1.512286949 | 6.94E-169 | 1.1627295 | 6.88E-41 | 2.20E-170 | 6.73E-42 | 1.345489529 | 1.58E-210 | 4.05E-209 |
| 259266 | 'ASPM' | -1.681177816 | 1.25E-91 | -1.818759685 | 1.16E-41 | 6.91E-93 | 1.12E-42 | -1.842010385 | 1.03E-132 | 1.71E-131 |
| 26084 | 'ARHGEF26' | -1.160991877 | 6.43E-12 | -1.099535674 | 1.66E-18 | 1.75E-12 | 3.03E-19 | -1.086484419 | 1.96E-30 | 9.99E-30 |
| 26150 | 'RIBC2' | -2.820178962 | 6.74E-63 | -2.94753258 | 7.56E-43 | 5.24E-64 | 7.12E-44 | -2.872325036 | 5.15E-105 | 6.89E-104 |
| 26232 | 'FBXO2' | 1.016944141 | 9.88E-292 | 2.92353658 | 0 | 1.72E-293 | 0 | 1.255788447 | 0 | 0 |
| 26271 | 'FBXO5' | -2.944923728 | 8.98E-226 | -2.343301746 | 2.90E-161 | 2.12E-227 | 7.33E-163 | -2.620748002 | 0 | 0 |
| 26470 | 'SEZ6L2' | 1.113448823 | 0 | 1.183793305 | 0 | 0 | 0 | 1.135930079 | 0 | 0 |
| 26586 | 'CKAP2' | -1.564671097 | 3.97E-80 | -1.244395822 | 7.46E-56 | 2.47E-81 | 5.56E-57 | -1.385012348 | 2.65E-135 | 4.47E-134 |
| 2668 | 'GDNF' | -2.102361718 | 5.35E-09 | -2.146841388 | 1.32E-04 | 1.70E-09 | 5.31E-05 | -2.04412977 | 3.03E-13 | 9.32E-13 |
| 267004 | 'PGBD3' | 2.879145605 | 9.76E-12 | 4.95419631 | 4.58E-06 | 2.69E-12 | 1.60E-06 | 3.250591081 | 1.97E-17 | 7.06E-17 |
| 2678 | 'GGT1' | 1.204013892 | 6.63E-14 | 1.327804661 | 1.59E-06 | 1.65E-14 | 5.35E-07 | 1.262080812 | 4.02E-20 | 1.58E-19 |
| 26973 | 'CHORDC1' | -1.306816098 | 8.48E-84 | -1.077722295 | 1.40E-25 | 5.05E-85 | 2.04E-26 | -1.248569687 | 4.27E-108 | 5.85E-107 |

| | | | | | | | | | | |
|---|---|---|---|---|---|---|---|---|---|---|
| 27124 | 'INPP5J' | 1.033166864 | 6.79E-05 | 1.911066272 | 5.91E-16 | 2.88E-05 | 1.19E-16 | 1.559656605 | 1.38E-19 | 5.31E-19 |
| 27338 | 'UBE2S' | -1.452558854 | 0 | -1.960076722 | 0 | 0 | 0 | -1.611182753 | 0 | 0 |
| 27344 | 'PCSK1N' | 1.758247157 | 1.71E-292 | 1.065691342 | 2.29E-30 | 2.98E-294 | 2.92E-31 | 1.604859491 | 0 | 0 |
| 27346 | 'TMEM97' | -1.427326756 | 2.71E-129 | -1.361707997 | 3.00E-66 | 1.12E-130 | 1.91E-67 | -1.40365321 | 9.29E-195 | 2.20E-193 |
| 2788 | 'GNG7' | 1.145605322 | 2.49E-15 | 1.36923381 | 4.44E-08 | 5.83E-16 | 1.35E-08 | 1.200379504 | 2.53E-23 | 1.09E-22 |
| 28231 | 'SLCO4A1' | 1.232660757 | 2.85E-55 | -1.663636815 | 1.89E-30 | 2.47E-56 | 2.41E-31 | 0.560277856 | 2.03E-19 | 7.77E-19 |
| 283431 | 'GAS2L3' | -2.381249186 | 4.90E-80 | -1.429812852 | 4.04E-49 | 3.06E-81 | 3.39E-50 | -1.789747509 | 4.65E-125 | 7.31E-124 |
| 283987 | 'HID1' | 2.106915204 | 5.01E-07 | 1.526898526 | 6.69E-35 | 1.80E-07 | 7.55E-36 | 1.554866246 | 6.10E-41 | 3.88E-40 |
| 284071 | 'MEIOC' | -1.064130337 | 2.16E-06 | -1.10780329 | 2.98E-16 | 8.12E-07 | 5.90E-17 | -1.105159204 | 1.22E-22 | 5.17E-22 |
| 284297 | 'SSC5D' | 2.045642666 | 3.11E-46 | 2.608046114 | 5.45E-41 | 3.16E-47 | 5.32E-42 | 2.240298138 | 1.29E-86 | 1.49E-85 |
| 284403 | 'WDR62' | -1.225748829 | 2.16E-147 | -1.545682841 | 0 | 7.90E-149 | 0 | -1.467029538 | 0 | 0 |
| 286151 | 'FBXO43' | -2.91020333 | 1.58E-35 | -1.641993446 | 1.55E-29 | 2.02E-36 | 2.01E-30 | -2.025815773 | 7.06E-62 | 6.25E-61 |
| 2896 | 'GRN' | 1.136605833 | 0 | 1.069909043 | 0 | 0 | 0 | 1.102891236 | 0 | 0 |
| 29028 | 'ATAD2' | -2.032782332 | 0 | -1.177970115 | 3.35E-82 | 0 | 1.76E-83 | -1.732775625 | 0 | 0 |
| 29089 | 'UBE2T' | -1.586836739 | 4.53E-171 | -1.966385122 | 9.95E-145 | 1.42E-172 | 2.81E-146 | -1.729832339 | 0 | 0 |
| 29127 | 'RACGAP1' | -1.883048477 | 0 | -1.031324111 | 2.57E-224 | 0 | 4.49E-226 | -1.365134695 | 0 | 0 |
| 29128 | 'UHRF1' | -2.484315352 | 0 | -1.454183829 | 6.20E-287 | 0 | 8.01E-289 | -1.765214184 | 0 | 0 |
| 29781 | 'NCAPH2' | -1.017707055 | 2.30E-74 | -1.200824268 | 1.05E-117 | 1.53E-75 | 3.69E-119 | -0.979036317 | 9.78E-189 | 2.25E-187 |
| 29893 | 'PSMC3IP' | -1.725220386 | 3.21E-39 | -1.546488353 | 9.90E-24 | 3.80E-40 | 1.52E-24 | -1.645102183 | 1.78E-62 | 1.58E-61 |
| 29899 | 'GPSM2' | -1.548620654 | 7.41E-101 | -1.772912291 | 1.44E-57 | 3.76E-102 | 1.05E-58 | -1.478667023 | 7.53E-158 | 1.47E-156 |
| 30008 | 'EFEMP2' | 1.673041659 | 1.20E-64 | 1.400607543 | 2.96E-62 | 9.09E-66 | 2.00E-63 | 1.506128137 | 5.22E-126 | 8.27E-125 |
| 3014 | 'H2AFX' | -2.025276799 | 0 | -1.950084856 | 0 | 0 | 0 | -1.988115967 | 0 | 0 |
| 3015 | 'H2AFZ' | -1.134472878 | 0 | -1.362479803 | 0 | 0 | 0 | -1.2523621 | 0 | 0 |
| 3070 | 'HELLS' | -1.433676453 | 5.63E-126 | -1.202242722 | 7.33E-78 | 2.39E-127 | 4.06E-79 | -1.363022654 | 1.53E-203 | 3.81E-202 |
| 3108 | 'HLA-DMA' | 1.229012198 | 4.57E-19 | 3.08246216 | 3.44E-38 | 9.22E-20 | 3.57E-39 | 1.689402692 | 2.03E-49 | 1.49E-48 |
| 3113 | 'HLA-DPA1' | 1.752503163 | 7.88E-23 | 1.827819025 | 3.14E-10 | 1.39E-23 | 8.36E-11 | 1.769309914 | 3.76E-33 | 2.04E-32 |
| 3134 | 'HLA-F' | 1.720369821 | 1.65E-69 | 1.248623963 | 5.16E-11 | 1.16E-70 | 1.32E-11 | 1.524699835 | 2.25E-79 | 2.41E-78 |
| 3140 | 'MR1' | 1.089981367 | 2.11E-22 | 2.793549123 | 3.91E-76 | 3.78E-23 | 2.22E-77 | 1.698814174 | 2.83E-84 | 3.17E-83 |
| 3146 | 'HMGB1' | -1.317817128 | 0 | -1.963695502 | 0 | 0 | 0 | -1.64108401 | 0 | 0 |
| 3148 | 'HMGB2' | -2.350225066 | 0 | -2.521873493 | 0 | 0 | 0 | -2.427308724 | 0 | 0 |
| 3149 | 'HMGB3' | -1.164511505 | 2.77E-81 | -1.469290259 | 2.91E-164 | 1.69E-82 | 7.11E-166 | -1.340756427 | 1.53E-244 | 4.65E-243 |
| 3151 | 'HMGN2' | -1.623375501 | 0 | -1.097492667 | 0 | 0 | 0 | -1.332778682 | 0 | 0 |
| 3161 | 'HMMR' | -2.822541326 | 7.68E-111 | -1.941979047 | 6.28E-86 | 3.59E-112 | 3.19E-87 | -2.277641146 | 1.80E-192 | 4.23E-191 |
| 3181 | 'HNRNPA2B1' | -1.437777551 | 0 | -1.368329145 | 0 | 0 | 0 | -1.36735146 | 0 | 0 |
| 3184 | 'HNRNPD' | -1.225583466 | 0 | -1.256088429 | 0 | 0 | 0 | -1.238727801 | 0 | 0 |
| 3189 | 'HNRNPH3' | -1.133327848 | 9.77E-194 | -1.291371046 | 1.37E-201 | 2.74E-195 | 2.76E-203 | -1.208338995 | 0 | 0 |
| 3196 | 'TLX2' | 1.173331603 | 3.13E-04 | 2.874469118 | 4.09E-05 | 1.41E-04 | 1.56E-05 | 1.505082705 | 4.01E-08 | 9.76E-08 |
| 332 | 'BIRC5' | -2.480621434 | 0 | -1.800833915 | 0 | 0 | 0 | -2.118631907 | 0 | 0 |
| 3320 | 'HSP90AA1' | -1.260106354 | 0 | -1.044036796 | 0 | 0 | 0 | -1.118326492 | 0 | 0 |
| 3339 | 'HSPG2' | 1.120355213 | 0 | 1.288201938 | 0 | 0 | 0 | 1.174078298 | 0 | 0 |
| 3383 | 'ICAM1' | 1.944043594 | 1.33E-204 | 1.255399527 | 9.64E-199 | 3.51E-206 | 1.96E-200 | 1.452075983 | 0 | 0 |
| 338707 | 'B4GALNT4' | 1.208979704 | 5.69E-60 | 1.078877536 | 7.98E-33 | 4.60E-61 | 9.49E-34 | 1.163654775 | 2.90E-93 | 3.54E-92 |
| 339122 | 'RAB43' | 1.415037499 | 2.18E-160 | 1.154161202 | 3.15E-67 | 7.26E-162 | 1.98E-68 | 1.282627324 | 8.67E-228 | 2.43E-226 |
| 339541 | 'ARMH1' | 2.014950341 | 5.54E-06 | 2.328325866 | 4.81E-14 | 2.15E-06 | 1.05E-14 | 2.204328633 | 1.11E-19 | 4.30E-19 |
| 340061 | 'TMEM173' | 1.588825445 | 0 | 1.273421885 | 8.82E-129 | 0 | 2.81E-130 | 1.521387633 | 0 | 0 |
| 3425 | 'IDUA' | 1.784111099 | 1.89E-114 | 1.569941404 | 1.70E-50 | 8.63E-116 | 1.38E-51 | 1.67183599 | 1.21E-165 | 2.47E-164 |
| 3426 | 'CFI' | 1.711874613 | 1.26E-05 | 5.459431619 | 1.97E-08 | 5.02E-06 | 5.83E-09 | 2.232710435 | 8.86E-12 | 2.57E-11 |
| 3429 | 'IFI27' | 1.771565785 | 1.91E-244 | 2.640003864 | 1.68E-12 | 4.11E-246 | 3.98E-13 | 1.802985023 | 1.47E-260 | 4.78E-259 |
| 342918 | 'C19orf81' | 1.631693551 | 8.23E-05 | -1.146841388 | 1.97E-04 | 3.51E-05 | 8.05E-05 | -0.125565415 | 0.570653964 | 0.638006878 |
| 3433 | 'IFIT2' | 1.88278705 | 0 | 1.99138687 | 1.99E-36 | 0 | 2.16E-37 | 1.897670998 | 0 | 0 |
| 3434 | 'IFIT1' | 1.551852323 | 0 | 2.799087306 | 3.22E-107 | 0 | 1.28E-108 | 1.660840733 | 0 | 0 |
| 3437 | 'IFIT3' | 1.785237734 | 0 | 1.854746977 | 6.73E-40 | 0 | 6.73E-41 | 1.793689648 | 0 | 0 |
| 348 | 'APOE' | 1.90802933 | 1.21E-73 | 3.016316661 | 0 | 8.05E-75 | 0 | 2.871051661 | 0 | 0 |
| 348654 | 'GEN1' | -1.429137696 | 4.38E-46 | -1.067114196 | 4.16E-16 | 4.46E-47 | 8.30E-17 | -1.327084813 | 3.18E-61 | 2.78E-60 |
| 348738 | 'C2orf48' | -3.662965013 | 2.73E-13 | -5.321928095 | 8.82E-07 | 6.99E-14 | 2.91E-07 | -4.060071314 | 2.09E-19 | 7.98E-19 |
| 3619 | 'INCENP' | -2.058194878 | 3.05E-286 | -1.120861849 | 7.88E-121 | 5.39E-288 | 2.65E-122 | -1.469729477 | 0 | 0 |
| 3669 | 'ISG20' | 1.672089111 | 6.69E-179 | 1.416737788 | 9.42E-16 | 2.04E-180 | 1.91E-16 | 1.602142335 | 2.37E-197 | 5.73E-196 |
| 3726 | 'JUNB' | 1.049365224 | 0 | 1.311434438 | 0 | 0 | 0 | 1.153244985 | 0 | 0 |
| 374383 | 'NCR3LG1' | -1.736965594 | 7.47E-11 | -1.152951923 | 1.87E-16 | 2.15E-11 | 3.67E-17 | -1.300282076 | 1.01E-26 | 4.74E-26 |
| 374393 | 'FAM111B' | -5.276124405 | 1.16E-124 | -3.991779493 | 2.97E-40 | 5.01E-126 | 2.95E-41 | -4.792736771 | 3.41E-154 | 6.51E-153 |
| 374977 | 'MROH7' | 3.722466024 | 4.96E-19 | 1.91753784 | 2.61E-05 | 1.00E-19 | 9.78E-06 | 2.873816014 | 9.67E-23 | 4.11E-22 |
| 375775 | 'PNPLA7' | 1.621488377 | 1.02E-14 | 2 | 3.44E-22 | 2.46E-15 | 5.48E-23 | 1.810019363 | 2.29E-36 | 1.34E-35 |
| 375790 | 'AGRN' | 1.047694409 | 0 | 1.38554903 | 0 | 0 | 0 | 1.20206159 | 0 | 0 |
| 3761 | 'KCNJ4' | 1.710493383 | 8.13E-05 | 3.232660757 | 1.03E-05 | 3.47E-05 | 3.73E-06 | 2.211951875 | 7.78E-10 | 2.07E-09 |
| 3780 | 'KCNN1' | 1.543666084 | 2.10E-52 | 2.305897985 | 4.58E-76 | 1.92E-53 | 2.60E-77 | 1.822584707 | 1.26E-124 | 1.97E-123 |
| 3832 | 'KIF11' | -2.428995957 | 1.19E-295 | -1.713695815 | 1.46E-116 | 2.06E-297 | 5.21E-118 | -2.111743489 | 0 | 0 |
| 3833 | 'KIFC1' | -2.77226061 | 2.74E-300 | -1.909610097 | 0 | 4.72E-302 | 0 | -2.076177242 | 0 | 0 |
| 3838 | 'KPNA2' | -1.402506808 | 0 | -1.218077145 | 0 | 0 | 0 | -1.305064431 | 0 | 0 |
| 387103 | 'CENPW' | -1.02289084 | 2.84E-30 | -1.116956146 | 1.29E-34 | 4.07E-31 | 1.47E-35 | -1.077144828 | 7.09E-65 | 6.47E-64 |
| 387522 | 'TMEM189-UBE2 | -1.969626351 | 3.40E-05 | 1.662965013 | 4.68E-05 | 1.40E-05 | 1.80E-05 | 0.09598318 | 0.721479624 | 0.774280851 |
| 388341 | 'LRRC75A' | -1.056583528 | 1.71E-04 | 1.371968777 | 1.28E-34 | 7.50E-05 | 1.45E-35 | 0.974517292 | 5.36E-23 | 2.29E-22 |
| 388403 | 'YPEL2' | 1.029146346 | 3.85E-07 | 1.40541308 | 1.78E-25 | 1.37E-07 | 2.59E-26 | 1.278685864 | 1.11E-31 | 5.84E-31 |
| 388588 | 'SMIM1' | 1.59946207 | 1.23E-04 | 1.974939079 | 1.34E-20 | 5.36E-05 | 2.27E-21 | 1.857118159 | 1.06E-24 | 4.73E-24 |
| 3902 | 'LAG3' | 1.05626822 | 5.02E-05 | -2 | 1.67E-08 | 2.10E-05 | 4.93E-09 | -0.036987701 | 0.841303875 | 0.879686611 |
| 3913 | 'LAMB2' | 1.300761892 | 0 | 1.716559384 | 0 | 0 | 0 | 1.403283599 | 0 | 0 |
| 3925 | 'STMN1' | -1.514829996 | 0 | -1.056028226 | 0 | 0 | 0 | -1.295132886 | 0 | 0 |
| 3930 | 'LBR' | -1.878041621 | 4.59E-133 | -1.020671079 | 7.63E-53 | 1.83E-134 | 5.97E-54 | -1.396995133 | 4.71E-173 | 9.88E-172 |
| 3959 | 'LGALS3BP' | 1.499299519 | 0 | 2.418905798 | 0 | 0 | 0 | 1.67882975 | 0 | 0 |

| | | | | | | | | | | |
|---|---|---|---|---|---|---|---|---|---|---|
| 3978 | 'LIG1' | -1.200460938 | 2.03E-121 | -1.349842617 | 4.80E-119 | 8.90E-123 | 1.65E-120 | -1.263758563 | 7.69E-240 | 2.30E-238 |
| 398 | 'ARHGDIG' | 1.748587514 | 6.87E-11 | 1.653771659 | 3.57E-08 | 1.97E-11 | 1.08E-08 | 1.698374007 | 8.58E-19 | 3.22E-18 |
| 399668 | 'SMIM10L2A' | 1.222392421 | 1.16E-07 | 2.025535092 | 1.25E-10 | 3.97E-08 | 3.27E-11 | 1.494439825 | 3.78E-17 | 1.34E-16 |
| 4001 | 'LMNB1' | -3.479435414 | 0 | -2.132916254 | 0 | 0 | 0 | -2.431858575 | 0 | 0 |
| 402778 | 'IFITM10' | 1.643537327 | 1.04E-99 | 2.280107919 | 2.05E-10 | 5.36E-101 | 5.40E-11 | 1.686857117 | 1.62E-111 | 2.29E-110 |
| 4035 | 'LRP1' | 1.168246295 | 0 | 2.127941183 | 0 | 0 | 0 | 1.402268896 | 0 | 0 |
| 4059 | 'BCAM' | 1.357107989 | 0 | 1.640367296 | 0 | 0 | 0 | 1.464730623 | 0 | 0 |
| 4085 | 'MAD2L1' | -1.872469394 | 8.25E-178 | -1.141698085 | 1.43E-35 | 2.52E-179 | 1.59E-36 | -1.624514796 | 1.21E-204 | 3.03E-203 |
| 410 | 'ARSA' | 1.211694847 | 3.10E-112 | 1.327203037 | 1.51E-33 | 1.43E-113 | 1.77E-34 | 1.174605414 | 5.07E-148 | 9.31E-147 |
| 4125 | 'MAN2B1' | 1.1019286 | 2.09E-88 | 1.191161282 | 7.74E-224 | 1.19E-89 | 1.37E-225 | 1.152737476 | 0 | 0 |
| 4137 | 'MAPT' | 1.089637212 | 1.10E-07 | 1.168787678 | 4.93E-27 | 3.78E-08 | 6.88E-28 | 1.155570772 | 6.01E-34 | 3.34E-33 |
| 4148 | 'MATN3' | 1.121344821 | 5.54E-23 | 1.356693513 | 9.64E-06 | 9.71E-24 | 3.47E-06 | 1.161482087 | 6.21E-29 | 3.07E-28 |
| 4171 | 'MCM2' | -1.953429589 | 0 | -1.142776423 | 0 | 0 | 0 | -1.454444011 | 0 | 0 |
| 4173 | 'MCM4' | -2.072209986 | 0 | -1.410679061 | 0 | 0 | 0 | -1.619377153 | 0 | 0 |
| 4175 | 'MCM6' | -1.834843359 | 0 | -1.471492916 | 0 | 0 | 0 | -1.615496856 | 0 | 0 |
| 4176 | 'MCM7' | -1.643824076 | 0 | -1.983736697 | 0 | 0 | 0 | -1.889994084 | 0 | 0 |
| 4288 | 'MKI67' | -3.398766033 | 0 | -2.335965928 | 0 | 0 | 0 | -2.860302933 | 0 | 0 |
| 4312 | 'MMP1' | -1.458214463 | 8.40E-178 | 2.123382416 | 2.63E-37 | 2.57E-179 | 2.80E-38 | -1.072081332 | 1.64E-113 | 2.36E-112 |
| 4320 | 'MMP11' | 1.597901556 | 1.84E-12 | 2.156947147 | 2.73E-114 | 4.87E-13 | 9.94E-116 | 2.055832673 | 1.71E-124 | 2.67E-123 |
| 440145 | 'MZT1' | -1.09592442 | 9.09E-22 | -1.403632736 | 8.67E-31 | 1.66E-22 | 1.10E-31 | -1.243671253 | 7.54E-52 | 5.76E-51 |
| 4436 | 'MSH2' | -1.580990867 | 8.79E-108 | -1.113426061 | 7.59E-36 | 4.19E-109 | 8.38E-37 | -1.408256906 | 1.11E-139 | 1.93E-138 |
| 4605 | 'MYBL2' | -2.580014761 | 0 | -2.109079033 | 0 | 0 | 0 | -2.358658379 | 0 | 0 |
| 4751 | 'NEK2' | -3.06196816 | 3.47E-198 | -1.28368426 | 1.71E-39 | 9.39E-200 | 1.73E-40 | -2.119536494 | 1.70E-209 | 4.34E-208 |
| 4778 | 'NFE2' | 1.444001578 | 4.51E-33 | 2.613531653 | 1.89E-11 | 6.02E-34 | 4.73E-12 | 1.562172202 | 1.33E-43 | 8.89E-43 |
| 4854 | 'NOTCH3' | 1.143402377 | 0 | 2.755301653 | 0 | 0 | 0 | 1.366060542 | 0 | 0 |
| 4858 | 'NOVA2' | 1.011777168 | 2.15E-22 | 1.499964848 | 3.45E-61 | 3.85E-23 | 2.38E-62 | 1.281237676 | 5.45E-81 | 5.98E-80 |
| 489 | 'ATP2A3' | 1.046542586 | 5.32E-08 | 1.173648087 | 8.06E-14 | 1.78E-08 | 1.78E-14 | 1.107089903 | 2.39E-21 | 9.72E-21 |
| 4900 | 'NRGN' | -1.812146727 | 3.18E-154 | -1.371488719 | 5.73E-59 | 1.11E-155 | 4.06E-60 | -1.643020746 | 7.23E-210 | 1.85E-208 |
| 4938 | 'OAS1' | 1.83162879 | 2.04E-215 | 4.108524457 | 1.33E-71 | 5.12E-217 | 7.95E-73 | 1.923256551 | 1.02E-275 | 3.49E-274 |
| 4939 | 'OAS2' | 1.360947001 | 4.08E-281 | 5.26052755 | 4.26E-97 | 7.36E-283 | 1.88E-98 | 1.774243752 | 0 | 0 |
| 494143 | 'CHAC2' | -1.119111212 | 1.81E-05 | -1.324801037 | 3.01E-17 | 7.29E-06 | 5.75E-18 | -1.28513787 | 1.32E-22 | 5.58E-22 |
| 494514 | 'TYMSOS' | -1.552541023 | 4.04E-06 | -1.839691896 | 5.21E-21 | 1.55E-06 | 8.62E-22 | -1.789383336 | 4.19E-27 | 1.99E-26 |
| 4987 | 'OPRL1' | 1.364807163 | 4.73E-27 | 1.524997712 | 2.69E-27 | 7.38E-28 | 3.73E-28 | 1.419430679 | 2.95E-54 | 2.33E-53 |
| 4998 | 'ORC1' | -2.351046427 | 1.26E-126 | -1.835924074 | 4.52E-94 | 5.33E-128 | 2.07E-95 | -2.075872209 | 1.20E-218 | 3.19E-217 |
| 50944 | 'SHANK1' | 1.593090382 | 2.58E-79 | 1.12225575 | 3.07E-42 | 1.62E-80 | 2.93E-43 | 1.348208162 | 1.01E-118 | 1.50E-117 |
| 51053 | 'GMNN' | -1.752072487 | 2.98E-65 | -2.448797102 | 1.58E-230 | 2.23E-66 | 2.69E-232 | -2.230798036 | 1.62E-292 | 5.97E-291 |
| 51090 | 'PLLP' | 1.038889013 | 3.25E-07 | 1.141245925 | 1.37E-15 | 1.15E-07 | 2.80E-16 | 1.094382278 | 2.77E-22 | 1.16E-21 |
| 5111 | 'PCNA' | -2.004508606 | 0 | -2.00719224 | 0 | 0 | 0 | -2.00614263 | 0 | 0 |
| 51110 | 'LACTB2' | -1.037010571 | 7.03E-21 | -1.012163309 | 1.18E-36 | 1.32E-21 | 1.27E-37 | -1.03326513 | 4.38E-58 | 3.67E-57 |
| 51129 | 'ANGPTL4' | 1.281797411 | 0 | 1.0040994 | 6.79E-49 | 0 | 5.72E-50 | 1.253239043 | 0 | 0 |
| 51148 | 'CERCAM' | 1.049358765 | 0 | 1.218901559 | 0 | 0 | 0 | 1.095402525 | 0 | 0 |
| 51200 | 'CPA4' | 1.753460027 | 0 | 2.842592102 | 1.05E-49 | 0 | 8.75E-51 | 1.784751917 | 0 | 0 |
| 51203 | 'NUSAP1' | -2.696305208 | 0 | -1.774183231 | 0 | 0 | 0 | -2.226662501 | 0 | 0 |
| 51279 | 'C1RL' | 1.110817991 | 1.13E-49 | 1.556393349 | 1.14E-41 | 1.08E-50 | 1.09E-42 | 1.293917748 | 6.92E-90 | 8.15E-89 |
| 5129 | 'CDK18' | 1.233363539 | 3.81E-44 | 2.519028231 | 9.06E-72 | 4.06E-45 | 5.40E-73 | 1.580569731 | 1.81E-105 | 2.44E-104 |
| 51296 | 'SLC15A3' | 1.807554038 | 2.07E-203 | 5.044394119 | 2.34E-06 | 5.47E-205 | 7.94E-07 | 1.828580622 | 5.90E-212 | 1.53E-210 |
| 51310 | 'SLC22A17' | 1.209062091 | 1.12E-93 | 1.11117891 | 5.98E-24 | 6.06E-95 | 9.09E-25 | 1.181288392 | 2.13E-119 | 3.20E-118 |
| 51512 | 'GTSE1' | -2.644072308 | 0 | -1.687236019 | 1.30E-289 | 0 | 1.67E-291 | -2.036677092 | 0 | 0 |
| 51514 | 'DTL' | -2.342271958 | 1.04E-278 | -1.019705409 | 5.79E-51 | 1.92E-280 | 4.70E-52 | -1.699874588 | 4.72E-293 | 1.74E-291 |
| 5159 | 'PDGFRB' | 2.169925001 | 9.59E-21 | 1.765534746 | 9.17E-06 | 1.82E-21 | 3.30E-06 | 2.035853106 | 2.77E-26 | 1.28E-25 |
| 51659 | 'GINS2' | -2.675170277 | 1.24E-226 | -2.200088709 | 0 | 2.92E-228 | 0 | -2.365217957 | 0 | 0 |
| 51702 | 'PADI3' | -2.408805546 | 7.04E-21 | 1.162271429 | 2.34E-05 | 1.33E-21 | 8.72E-06 | -0.672491811 | 7.45E-05 | 1.48E-04 |
| 5265 | 'SERPINA1' | 1.953349907 | 1.54E-80 | 1.307385537 | 2.81E-123 | 9.52E-82 | 9.33E-125 | 1.443818041 | 1.43E-196 | 3.42E-195 |
| 53340 | 'SPA17' | -1.191719977 | 9.04E-40 | -1.162938571 | 3.97E-29 | 1.06E-40 | 5.21E-30 | -1.184120346 | 8.59E-69 | 8.23E-68 |
| 53371 | 'NUP54' | -1.16801995 | 1.27E-62 | -1.041340795 | 4.21E-43 | 9.91E-64 | 3.95E-44 | -1.106167729 | 1.49E-105 | 2.01E-104 |
| 5347 | 'PLK1' | -2.581192351 | 0 | -2.075623234 | 0 | 0 | 0 | -2.31834419 | 0 | 0 |
| 53822 | 'FXYD7' | 1.310340121 | 4.65E-06 | 2.038135129 | 2.97E-05 | 1.79E-06 | 1.12E-05 | 1.497983313 | 1.16E-10 | 3.21E-10 |
| 5393 | 'EXOSC9' | -1.026174883 | 2.10E-42 | -1.325109342 | 5.09E-31 | 2.33E-43 | 6.40E-32 | -1.12292163 | 4.92E-72 | 4.91E-71 |
| 5427 | 'POLE2' | -1.886302014 | 2.09E-52 | -1.106915204 | 3.03E-19 | 1.91E-53 | 5.39E-20 | -1.497761397 | 5.52E-68 | 5.24E-67 |
| 5433 | 'POLR2D' | -1.017115421 | 2.68E-64 | -1.127723211 | 3.29E-252 | 2.04E-65 | 5.02E-254 | -1.114780082 | 0 | 0 |
| 54443 | 'ANLN' | -1.956977985 | 0 | -1.441189787 | 3.04E-219 | 0 | 5.55E-221 | -1.829838573 | 0 | 0 |
| 54478 | 'PIMREG' | -2.079136909 | 1.83E-225 | -2.091423028 | 9.57E-67 | 4.33E-227 | 6.04E-68 | -2.082095931 | 4.58E-290 | 1.66E-288 |
| 54492 | 'NEURL1B' | -3.584962501 | 3.06E-71 | -2.567684509 | 1.37E-165 | 2.11E-72 | 3.34E-167 | -2.747156439 | 9.94E-235 | 2.87E-233 |
| 54507 | 'ADAMTSL4' | 1.792579569 | 1.22E-197 | 1.36767426 | 7.10E-94 | 3.31E-199 | 3.27E-95 | 1.499405433 | 1.14E-289 | 4.13E-288 |
| 54734 | 'RAB39A' | -1.963474124 | 2.50E-07 | -1.505235308 | 2.49E-04 | 8.76E-08 | 1.03E-04 | -1.757866311 | 3.56E-11 | 1.00E-10 |
| 54821 | 'ERCC6L' | -2.028196892 | 1.17E-63 | -1.269186633 | 1.21E-32 | 8.94E-65 | 1.45E-33 | -1.59297631 | 2.36E-92 | 2.85E-91 |
| 54908 | 'SPDL1' | -1.070463276 | 1.43E-71 | -1.020553811 | 1.24E-89 | 9.81E-73 | 6.02E-91 | -1.079021138 | 1.92E-162 | 3.85E-161 |
| 54932 | 'EXD3' | 1.533737177 | 1.69E-08 | 1.537396817 | 1.62E-18 | 5.52E-09 | 2.95E-19 | 1.565178482 | 1.31E-26 | 6.13E-26 |
| 54962 | 'TIPIN' | -1.418713157 | 1.79E-31 | -1.605665206 | 1.01E-32 | 2.50E-32 | 1.21E-33 | -1.343645165 | 5.28E-64 | 4.78E-63 |
| 54982 | 'CLN6' | -1.126067499 | 1.27E-79 | -1.241707832 | 1.02E-160 | 7.97E-81 | 2.59E-162 | -1.207880524 | 2.61E-242 | 7.83E-241 |
| 55038 | 'CDCA4' | -1.471346425 | 1.57E-235 | -1.065372874 | 0 | 3.51E-237 | 0 | -1.14665575 | 0 | 0 |
| 55071 | 'C9orf40' | -1.065033885 | 3.53E-21 | -1.231975702 | 6.61E-82 | 6.58E-22 | 3.49E-83 | -1.202548567 | 2.21E-104 | 2.94E-103 |
| 55143 | 'CDCA8' | -2.19758903 | 0 | -1.2238397 | 6.09E-220 | 0 | 1.11E-221 | -1.58599629 | 0 | 0 |
| 55165 | 'CEP55' | -2.259059255 | 2.45E-238 | -1.972191341 | 5.98E-169 | 5.42E-240 | 1.42E-170 | -2.127346624 | 0 | 0 |
| 55215 | 'FANCI' | -1.58813326 | 1.41E-196 | -1.270596284 | 2.34E-104 | 3.84E-198 | 9.61E-106 | -1.445346777 | 9.36E-298 | 3.50E-296 |
| 55247 | 'NEIL3' | -2.896906507 | 1.05E-194 | -2.314037309 | 9.09E-49 | 2.91E-196 | 7.67E-50 | -2.722398108 | 1.41E-239 | 4.19E-238 |
| 55281 | 'TMEM140' | 1.222392421 | 4.57E-19 | 2.012133756 | 7.36E-38 | 9.22E-20 | 7.71E-39 | 1.559976528 | 3.00E-54 | 2.37E-53 |
| 55320 | 'MIS18BP1' | -1.07570966 | 2.22E-32 | -1.062735755 | 2.60E-12 | 3.03E-33 | 6.22E-13 | -1.15681994 | 3.95E-44 | 2.65E-43 |

| | | | | | | | | | | |
|---|---|---|---|---|---|---|---|---|---|---|
| 55329 | 'MNS1' | -1.11311408 | 6.84E-09 | -2.8259706 | 4.80E-08 | 2.18E-09 | 1.46E-08 | -1.325232753 | 1.32E-14 | 4.31E-14 |
| 55355 | 'HJURP' | -2.802751891 | 0 | -1.971699857 | 0 | 0 | 0 | -2.270825197 | 0 | 0 |
| 55388 | 'MCM10' | -3.317970081 | 8.54E-184 | -1.841096405 | 7.82E-75 | 2.52E-185 | 4.52E-76 | -2.47850739 | 8.82E-244 | 2.68E-242 |
| 554282 | 'FAM72C' | -1.678798887 | 1.54E-23 | -2.031609607 | 1.35E-45 | 2.65E-24 | 1.20E-46 | -1.878881793 | 7.02E-69 | 6.74E-68 |
| 55558 | 'PLXNA3' | 1.029146346 | 1.87E-71 | 1.121650835 | 6.83E-115 | 1.29E-72 | 2.48E-116 | 1.094909819 | 1.56E-185 | 3.52E-184 |
| 5557 | 'PRIM1' | -3.144892853 | 1.22E-153 | -1.973193301 | 3.13E-113 | 4.28E-155 | 1.16E-114 | -2.394720207 | 8.75E-258 | 2.80E-256 |
| 55635 | 'DEPDC1' | -2.340682055 | 7.15E-196 | -1.234929068 | 9.26E-56 | 1.97E-197 | 6.91E-57 | -1.787295514 | 3.42E-233 | 9.76E-232 |
| 55679 | 'LIMS2' | 1.835658572 | 7.76E-51 | 1.234465254 | 1.91E-12 | 7.29E-52 | 4.52E-13 | 1.694388659 | 1.88E-62 | 1.67E-61 |
| 55698 | 'RADIL' | -1.131244533 | 1.52E-05 | -1.222392421 | 1.06E-10 | 6.11E-06 | 2.75E-11 | -1.190821859 | 5.52E-16 | 1.89E-15 |
| 55771 | 'PRR11' | -1.899113245 | 0 | -1.154183012 | 0 | 0 | 0 | -1.442455884 | 0 | 0 |
| 55789 | 'DEPDC1B' | -1.995353635 | 2.22E-93 | -1.074409605 | 1.05E-34 | 1.21E-94 | 1.19E-35 | -1.483221459 | 2.82E-119 | 4.22E-118 |
| 55839 | 'CENPN' | -1.411612844 | 1.33E-227 | -1.60083271 | 9.88E-180 | 3.12E-229 | 2.18E-181 | -1.172216657 | 0 | 0 |
| 55872 | 'PBK' | -2.268212458 | 4.75E-73 | -1.649092838 | 1.43E-41 | 3.20E-74 | 1.37E-42 | -1.968543219 | 1.51E-112 | 2.15E-111 |
| 55972 | 'SLC25A40' | -1.200000665 | 1.95E-29 | -1.17046102 | 7.69E-31 | 2.84E-30 | 9.71E-32 | -1.189391933 | 2.01E-60 | 1.74E-59 |
| 5602 | 'MAPK10' | 1.095157233 | 4.09E-05 | 1.03562391 | 1.42E-05 | 1.70E-05 | 5.19E-06 | 1.199018767 | 3.07E-10 | 8.32E-10 |
| 56126 | 'PCDHB10' | 1.070389328 | 3.26E-05 | 1.36983808 | 1.38E-12 | 1.34E-05 | 3.23E-13 | 1.245377871 | 4.90E-17 | 1.73E-16 |
| 56138 | 'PCDHA11' | 1.146841388 | 4.28E-07 | 1 | 4.22E-04 | 1.53E-07 | 1.79E-04 | 1.107206871 | 1.27E-10 | 3.49E-10 |
| 56154 | 'TEX15' | -1.906890596 | 6.80E-06 | -2.222392421 | 1.14E-06 | 2.66E-06 | 3.81E-07 | -2.034076106 | 3.39E-12 | 1.00E-11 |
| 56992 | 'KIF15' | -3.022026306 | 4.62E-114 | -1.669026766 | 1.10E-19 | 2.11E-115 | 1.93E-20 | -2.619654222 | 1.10E-128 | 1.77E-127 |
| 57082 | 'KNL1' | -2.276445325 | 1.35E-98 | -1.217417606 | 1.14E-28 | 7.00E-100 | 1.52E-29 | -1.741596363 | 1.57E-118 | 2.33E-117 |
| 57326 | 'PBXIP1' | 1.08660537 | 1.77E-133 | 2.059470208 | 0 | 7.02E-135 | 0 | 1.597173928 | 0 | 0 |
| 57405 | 'SPC25' | -3.028817272 | 1.17E-149 | -2.072134876 | 1.16E-101 | 4.18E-151 | 4.90E-103 | -2.473122142 | 1.40E-244 | 4.29E-243 |
| 57415 | 'C3orf14' | -1.027825261 | 1.66E-33 | -1.872567081 | 4.72E-35 | 2.19E-34 | 5.29E-36 | -1.246932024 | 4.09E-62 | 3.63E-61 |
| 57468 | 'SLC12A5' | 1.078900828 | 6.07E-28 | 1.365649472 | 3.20E-08 | 9.25E-29 | 9.64E-09 | 1.127994222 | 1.93E-36 | 1.12E-35 |
| 57478 | 'USP31' | -1.033251806 | 4.67E-28 | -1.068947354 | 2.06E-16 | 7.09E-29 | 4.05E-17 | -0.738726865 | 3.16E-43 | 2.09E-42 |
| 57524 | 'CASKIN1' | 1.015767316 | 2.07E-13 | 1.373458396 | 1.61E-13 | 5.26E-14 | 3.61E-14 | 1.191035967 | 3.47E-26 | 1.60E-25 |
| 57650 | 'CIP2A' | -2.048269209 | 1.90E-92 | -1.462432025 | 9.90E-30 | 1.04E-93 | 1.28E-30 | -1.67075638 | 1.56E-119 | 2.35E-118 |
| 57664 | 'PLEKHA4' | 1.958952251 | 5.34E-142 | 1.452512205 | 1.84E-12 | 2.01E-143 | 4.35E-13 | 1.789617903 | 4.31E-156 | 8.35E-155 |
| 57695 | 'USP37' | -1.533179959 | 2.47E-53 | -1.053111336 | 1.41E-16 | 2.21E-54 | 2.74E-17 | -1.336205747 | 1.03E-67 | 9.73E-67 |
| 57715 | 'SEMA4G' | 1.503805356 | 1.71E-50 | 1.593575631 | 1.00E-102 | 1.62E-51 | 4.16E-104 | 1.553351051 | 2.04E-152 | 3.83E-151 |
| 57731 | 'SPTBN4' | 1.181606806 | 9.33E-08 | 1.514227989 | 8.57E-71 | 3.18E-08 | 5.18E-72 | 1.56936314 | 2.05E-76 | 2.13E-75 |
| 57828 | 'CATSPERG' | 1.2410081 | 2.97E-04 | 1.398549376 | 1.81E-16 | 1.33E-04 | 3.54E-17 | 1.430498816 | 3.96E-20 | 1.55E-19 |
| 580 | 'BARD1' | -1.944422652 | 4.15E-57 | -1.980634675 | 1.31E-42 | 3.49E-58 | 1.24E-43 | -2.032703138 | 9.99E-100 | 1.28E-98 |
| 58538 | 'MPP4' | -1.265590111 | 9.98E-10 | -1.064342234 | 4.76E-22 | 3.04E-10 | 7.63E-23 | -1.093304618 | 5.70E-32 | 3.02E-31 |
| 5864 | 'RAB3A' | 1.229068486 | 8.34E-09 | 1.810175441 | 1.08E-10 | 2.68E-09 | 2.80E-11 | 1.436582768 | 1.02E-18 | 3.82E-18 |
| 5888 | 'RAD51' | -1.445761865 | 3.27E-57 | -1.667182038 | 5.41E-132 | 2.74E-58 | 1.68E-133 | -1.574644487 | 6.24E-191 | 1.46E-189 |
| 5920 | 'RARRES3' | 2.679446555 | 0 | 1.781359714 | 2.04E-21 | 0 | 3.33E-22 | 2.579194549 | 0 | 0 |
| 5933 | 'RBL1' | -2.03312207 | 6.21E-138 | -1.301002256 | 1.13E-31 | 2.40E-139 | 1.39E-32 | -1.765633008 | 7.17E-164 | 1.44E-162 |
| 596 | 'BCL2' | -1.520832163 | 1.18E-05 | -1.625604485 | 4.38E-09 | 4.69E-06 | 1.25E-09 | -1.49087613 | 2.14E-14 | 6.93E-14 |
| 5984 | 'RFC4' | -1.236800281 | 5.91E-63 | -1.437573704 | 1.01E-92 | 4.59E-64 | 4.69E-94 | -1.351852859 | 1.03E-155 | 1.98E-154 |
| 6240 | 'RRM1' | -1.762977663 | 1.42E-222 | -1.059092668 | 6.91E-95 | 3.41E-224 | 3.14E-96 | -1.39079567 | 3.50E-301 | 1.33E-299 |
| 6241 | 'RRM2' | -3.894595297 | 0 | -2.796475729 | 0 | 0 | 0 | -3.364503406 | 0 | 0 |
| 6275 | 'S100A4' | 1.044510201 | 5.45E-19 | 1.603955216 | 1.25E-09 | 1.10E-19 | 3.45E-10 | 1.129531172 | 4.65E-28 | 2.26E-27 |
| 634 | 'CEACAM1' | 2.263034406 | 1.37E-243 | 3.651051691 | 2.58E-41 | 2.97E-245 | 2.50E-42 | 2.484871254 | 4.18E-284 | 1.48E-282 |
| 63901 | 'FAM111A' | -1.901091641 | 8.39E-274 | -1.379754522 | 6.28E-62 | 1.59E-275 | 4.26E-63 | -1.721526098 | 0 | 0 |
| 63967 | 'CLSPN' | -2.25005486 | 8.59E-108 | -2.38932738 | 5.68E-109 | 4.09E-109 | 2.22E-110 | -2.263722752 | 3.00E-216 | 7.93E-215 |
| 63979 | 'FIGNL1' | -1.35474672 | 6.71E-110 | -1.222392421 | 1.32E-11 | 3.16E-111 | 3.26E-12 | -1.321160874 | 1.26E-119 | 1.90E-118 |
| 641 | 'BLM' | -1.660730008 | 1.43E-49 | -1.888453347 | 2.77E-31 | 1.37E-50 | 3.46E-32 | -1.858964658 | 1.15E-80 | 1.25E-79 |
| 64105 | 'CENPK' | -1.747353829 | 6.18E-71 | -1.715570083 | 4.25E-53 | 4.28E-72 | 3.31E-54 | -1.729005201 | 3.69E-124 | 5.75E-123 |
| 64151 | 'NCAPG' | -3.219623332 | 4.36E-286 | -2.394470164 | 4.33E-229 | 7.71E-288 | 7.43E-231 | -2.706576958 | 0 | 0 |
| 6421 | 'SFPQ' | -1.269570597 | 0 | -1.136352856 | 0 | 0 | 0 | -1.04308208 | 0 | 0 |
| 6426 | 'SRSF1' | -1.498374816 | 0 | -1.232194587 | 0 | 0 | 0 | -1.381977284 | 0 | 0 |
| 6427 | 'SRSF2' | -1.144056615 | 0 | -1.131385246 | 0 | 0 | 0 | -1.121861783 | 0 | 0 |
| 6428 | 'SRSF3' | -1.128635316 | 1.9856E-320 | -1.163536442 | 0 | 3.26E-322 | 0 | -1.152295901 | 0 | 0 |
| 6432 | 'SRSF7' | -1.056787058 | 1.77E-137 | -1.214712472 | 4.87E-188 | 6.84E-139 | 1.02E-189 | -1.031817716 | 0 | 0 |
| 6434 | 'TRA2B' | -1.025104415 | 2.76E-236 | -1.120580221 | 5.44E-265 | 6.16E-238 | 7.55E-267 | -1.109336581 | 0 | 0 |
| 647042 | 'GOLGA6L10' | -1.430634354 | 1.55E-06 | 2.070389328 | 3.80E-04 | 5.80E-07 | 1.60E-04 | -0.588440132 | 0.014292495 | 0.022581343 |
| 64798 | 'DEPTOR' | 1.427241639 | 8.64E-18 | 1.530514717 | 3.79E-18 | 1.84E-18 | 7.03E-19 | 1.507525974 | 1.38E-35 | 7.94E-35 |
| 649 | 'BMP1' | 1.24984048 | 6.08E-203 | 1.076530937 | 3.64E-66 | 1.61E-204 | 2.32E-67 | 1.135000107 | 1.61E-269 | 5.38E-268 |
| 652968 | 'CASTOR1' | 1.319521597 | 2.43E-32 | 1.433756697 | 5.83E-17 | 3.31E-33 | 1.13E-17 | 1.353294364 | 1.27E-49 | 9.36E-49 |
| 653820 | 'FAM72B' | -2.108993184 | 1.93E-73 | -1.682206363 | 5.90E-73 | 1.29E-74 | 3.46E-74 | -1.865842485 | 5.81E-146 | 1.05E-144 |
| 654 | 'BMP6' | 1.502500341 | 4.96E-05 | -1.023083613 | 2.33E-06 | 2.07E-05 | 7.90E-07 | -0.349266368 | 0.040605173 | 0.06020571 |
| 6590 | 'SLPI' | 1.637458806 | 0 | 2.362570079 | 8.39E-05 | 0 | 3.30E-05 | 1.635314629 | 0 | 0 |
| 6632 | 'SNRPD1' | -1.042891418 | 1.01E-122 | -1.072693662 | 7.47E-92 | 4.41E-124 | 3.52E-93 | -1.056678796 | 2.80E-214 | 7.31E-213 |
| 672 | 'BRCA1' | -2.038651879 | 9.00E-196 | -1.219242431 | 8.90E-28 | 2.48E-197 | 1.21E-28 | -1.896005286 | 9.93E-215 | 2.61E-213 |
| 6790 | 'AURKA' | -2.052800516 | 0 | -1.279227026 | 0 | 0 | 0 | -1.704662307 | 0 | 0 |
| 684 | 'BST2' | 1.548808672 | 0 | 4.857980995 | 1.80E-48 | 0 | 1.53E-49 | 1.600232412 | 0 | 0 |
| 6941 | 'TCF19' | -2.139235797 | 1.24E-217 | -2.050706962 | 6.32E-259 | 3.06E-219 | 9.11E-261 | -2.103587396 | 0 | 0 |
| 699 | 'BUB1' | -2.115040236 | 0 | -1.323701537 | 2.68E-254 | 0 | 4.06E-256 | -1.633243834 | 0 | 0 |
| 701 | 'BUB1B' | -2.502066186 | 0 | -1.070175294 | 1.55E-93 | 0 | 7.15E-95 | -1.651621307 | 0 | 0 |
| 7059 | 'THBS3' | 1.11318249 | 8.49E-45 | 1.339337773 | 2.14E-89 | 8.89E-46 | 1.04E-90 | 1.195215351 | 6.65E-133 | 1.10E-131 |
| 7083 | 'TK1' | -1.626913952 | 0 | -1.745553653 | 0 | 0 | 0 | -1.679226892 | 0 | 0 |
| 710 | 'SERPING1' | 1.629284832 | 1.78E-26 | 2.533201073 | 2.26E-95 | 2.82E-27 | 1.01E-96 | 2.164412786 | 1.13E-117 | 1.67E-116 |
| 7112 | 'TMPO' | -2.296922302 | 0 | -1.729272986 | 0 | 0 | 0 | -2.007188336 | 0 | 0 |
| 7148 | 'TNXB' | 1.807354922 | 1.73E-15 | 1.292180751 | 9.82E-16 | 4.03E-16 | 1.99E-16 | 1.222424234 | 7.88E-31 | 4.07E-30 |
| 715 | 'C1R' | 1.490033627 | 4.46E-202 | 1.353636955 | 3.13E-04 | 1.19E-203 | 1.31E-04 | 1.499237111 | 9.65E-211 | 2.48E-209 |
| 7153 | 'TOP2A' | -2.72066145 | 0 | -1.909950395 | 4.65E-300 | 0 | 5.73E-302 | -2.447221032 | 0 | 0 |
| 716 | 'C1S' | 1.786163499 | 0 | 1.506959989 | 4.76E-04 | 0 | 2.03E-04 | 1.794228513 | 0 | 0 |

| | | | | | | | | | | |
|---|---|---|---|---|---|---|---|---|---|---|
| 7161 | 'TP73' | -2.566346823 | 9.49E-68 | -1.402371087 | 1.33E-37 | 6.79E-69 | 1.40E-38 | -1.819756985 | 6.82E-99 | 8.74E-98 |
| 7263 | 'TST' | 1.050597433 | 2.71E-44 | 1.15548519 | 3.33E-36 | 2.89E-45 | 3.63E-37 | 1.080476529 | 6.57E-81 | 7.21E-80 |
| 727 | 'C5' | -1.869939459 | 8.04E-16 | -1.358453971 | 1.97E-11 | 1.85E-16 | 4.92E-12 | -1.574644487 | 1.00E-26 | 4.71E-26 |
| 7274 | 'TTPA' | -1.693896872 | 1.60E-04 | -1.733354341 | 6.53E-10 | 6.99E-05 | 1.78E-10 | -1.743326322 | 3.13E-14 | 1.00E-13 |
| 727800 | 'RNF208' | 1.051731163 | 1.44E-08 | 1.029317807 | 4.38E-42 | 4.67E-09 | 4.18E-43 | 1.009179793 | 1.30E-49 | 9.58E-49 |
| 728118 | 'NUTM2A' | 1.106915204 | 7.88E-06 | 1.656347134 | 5.16E-12 | 3.10E-06 | 1.25E-12 | 1.024620127 | 1.36E-16 | 4.74E-16 |
| 728833 | 'FAM72D' | -2.189553808 | 6.88E-55 | -1.744161096 | 3.63E-40 | 6.00E-56 | 3.61E-41 | -1.953181333 | 2.31E-94 | 2.84E-93 |
| 729533 | 'FAM72A' | -2.288676289 | 6.77E-69 | -1.339310173 | 3.21E-16 | 4.80E-70 | 6.37E-17 | -1.613528154 | 2.20E-77 | 2.32E-76 |
| 7298 | 'TYMS' | -3.018252632 | 0 | -2.644373416 | 0 | 0 | 0 | -2.800709691 | 0 | 0 |
| 7318 | 'UBA7' | 1.815961629 | 4.31E-124 | 2.653442239 | 4.24E-24 | 1.87E-125 | 6.41E-25 | 1.892481523 | 1.36E-148 | 2.51E-147 |
| 7373 | 'COL14A1' | 1.151886177 | 4.75E-162 | 3.321928095 | 3.65E-06 | 1.55E-163 | 1.27E-06 | 1.167859291 | 8.22E-171 | 1.71E-169 |
| 7398 | 'USP1' | -1.820676301 | 3.71E-161 | -1.911985001 | 1.83E-94 | 1.22E-162 | 8.34E-96 | -1.84129814 | 5.02E-255 | 1.59E-253 |
| 7448 | 'VTN' | 3.405566142 | 0 | 3.169925001 | 8.01E-04 | 0 | 3.49E-04 | 3.413530077 | 0 | 0 |
| 7468 | 'NSD2' | -1.82077864 | 0 | -1.368073555 | 0 | 0 | 0 | -1.506895645 | 0 | 0 |
| 7516 | 'XRCC2' | -2.052256237 | 1.23E-50 | -1.172946353 | 2.36E-11 | 1.16E-51 | 5.93E-12 | -1.707813812 | 1.18E-58 | 9.94E-58 |
| 7517 | 'XRCC3' | -1.133440265 | 1.17E-83 | -1.097937341 | 5.25E-203 | 6.98E-85 | 1.05E-204 | -1.115209477 | 4.09E-291 | 1.49E-289 |
| 7634 | 'ZNF80' | -2.115477217 | 5.77E-05 | 1.185303103 | 1.13E-19 | 2.43E-05 | 1.99E-20 | 0.936856113 | 1.04E-14 | 3.41E-14 |
| 770 | 'CA11' | 1.685402402 | 2.58E-45 | 1.588233919 | 6.09E-210 | 2.68E-46 | 1.17E-211 | 1.586095522 | 2.81E-252 | 8.75E-251 |
| 7840 | 'ALMS1' | -1.015596855 | 6.45E-29 | -1.029747343 | 2.01E-08 | 9.59E-30 | 5.97E-09 | -1.013320337 | 1.33E-36 | 7.79E-36 |
| 78987 | 'CRELD1' | 1.208081144 | 1.50E-67 | 1.170650156 | 5.76E-35 | 1.08E-68 | 6.49E-36 | 1.202252929 | 1.77E-103 | 2.35E-102 |
| 79019 | 'CENPM' | -2.31751618 | 2.62E-212 | -1.309835023 | 1.07E-44 | 6.66E-214 | 9.66E-46 | -1.908453926 | 1.34E-243 | 4.07E-242 |
| 79075 | 'DSCC1' | -1.4408335 | 3.23E-35 | -1.569855608 | 8.82E-43 | 4.14E-36 | 8.32E-44 | -1.510746057 | 3.87E-78 | 4.11E-77 |
| 7913 | 'DEK' | -1.44530016 | 1.12E-136 | -1.265598125 | 1.43E-175 | 4.35E-138 | 3.29E-177 | -1.341295081 | 0 | 0 |
| 79132 | 'DHX58' | 1.611004635 | 1.61E-63 | 2.432959407 | 1.69E-08 | 1.23E-64 | 4.99E-09 | 1.656034545 | 1.10E-72 | 1.10E-71 |
| 79148 | 'MMP28' | 2.415037499 | 1.57E-13 | 1.896164189 | 3.46E-07 | 3.98E-14 | 1.11E-07 | 2.193917952 | 1.58E-20 | 6.27E-20 |
| 79173 | 'C19orf57' | -1.060816001 | 1.97E-27 | -1.274251392 | 1.31E-36 | 3.05E-28 | 1.42E-37 | -1.170401303 | 3.92E-64 | 3.55E-63 |
| 79586 | 'CHPF' | 1.394240738 | 0 | 1.07326677 | 2.29E-183 | 0 | 4.90E-185 | 1.330319395 | 0 | 0 |
| 79621 | 'RNASEH2B' | -1.073581132 | 3.81E-15 | -1.107397791 | 6.36E-23 | 9.01E-16 | 9.93E-24 | -1.163373818 | 5.43E-38 | 3.27E-37 |
| 79682 | 'CENPU' | -2.284383726 | 1.08E-98 | -2.062735755 | 2.07E-46 | 5.57E-100 | 1.82E-47 | -2.202076196 | 1.30E-144 | 2.32E-143 |
| 79733 | 'E2F8' | -3.996074297 | 3.19E-85 | -2.572578776 | 2.56E-69 | 1.86E-86 | 1.57E-70 | -3.014287956 | 1.29E-149 | 2.40E-148 |
| 79805 | 'VASH2' | -1.534336428 | 1.14E-10 | -3.392317423 | 8.94E-06 | 3.32E-11 | 3.21E-06 | -1.891501593 | 1.93E-15 | 6.45E-15 |
| 79866 | 'BORA' | -1.302882339 | 4.10E-16 | -1.461511929 | 6.10E-21 | 9.31E-17 | 1.01E-21 | -1.404125949 | 1.25E-36 | 7.35E-36 |
| 79958 | 'DENND1C' | 2.285402219 | 6.02E-05 | 1.874469118 | 1.83E-09 | 2.54E-05 | 5.10E-10 | 1.986070467 | 5.98E-14 | 1.90E-13 |
| 79968 | 'WDR76' | -3.260619118 | 8.89E-281 | -1.924051147 | 3.83E-157 | 1.61E-282 | 9.93E-159 | -2.454526961 | 0 | 0 |
| 80010 | 'RMI1' | -1.698491446 | 1.68E-47 | -1.38364708 | 1.58E-25 | 1.67E-48 | 2.30E-26 | -1.555218989 | 2.06E-72 | 2.06E-71 |
| 80071 | 'CCDC15' | -1.204358499 | 1.55E-14 | -1.442943496 | 1.03E-06 | 3.74E-15 | 3.42E-07 | -1.208252697 | 1.28E-20 | 5.11E-20 |
| 80119 | 'PIF1' | -2.958314097 | 3.97E-81 | -2.147169087 | 6.76E-58 | 2.44E-82 | 4.86E-59 | -2.48975559 | 8.12E-137 | 1.39E-135 |
| 80179 | 'MYO19' | -1.14459169 | 1.82E-221 | -1.075267665 | 2.61E-119 | 4.38E-223 | 8.99E-121 | -1.243037823 | 0 | 0 |
| 8029 | 'CUBN' | -1.807354922 | 2.44E-04 | 1.351472371 | 9.17E-12 | 1.08E-04 | 2.26E-12 | 0.768243226 | 1.92E-06 | 4.23E-06 |
| 80320 | 'SP6' | 1.207407139 | 1.53E-72 | 1.394859617 | 5.40E-06 | 1.04E-73 | 1.90E-06 | 1.227334907 | 2.44E-80 | 2.65E-79 |
| 80781 | 'COL18A1' | 1.113786229 | 3.56E-92 | 1.080237114 | 1.17E-209 | 1.96E-93 | 2.27E-211 | 1.080069229 | 4.20E-299 | 1.58E-297 |
| 80833 | 'APOL3' | 1.512155677 | 7.98E-78 | 2.039528364 | 1.82E-08 | 5.10E-79 | 5.39E-09 | 1.489201995 | 1.71E-87 | 1.98E-86 |
| 81610 | 'FAM83D' | -1.88632258 | 0 | -1.604189879 | 9.46E-240 | 0 | 1.53E-241 | -1.766991412 | 0 | 0 |
| 81611 | 'ANP32E' | -1.764202178 | 4.98E-282 | -1.015596855 | 1.80E-57 | 8.91E-284 | 1.31E-58 | -1.523232491 | 0 | 0 |
| 81620 | 'CDT1' | -2.063127979 | 3.11E-224 | -1.53034591 | 0 | 7.43E-226 | 0 | -1.637233009 | 0 | 0 |
| 81794 | 'ADAMTS10' | 1.314201653 | 1.91E-42 | 1.584962501 | 4.14E-05 | 2.12E-43 | 1.58E-05 | 1.245363323 | 2.73E-48 | 1.97E-47 |
| 8208 | 'CHAF1B' | -1.803568319 | 4.28E-138 | -1.131244533 | 8.77E-81 | 1.65E-139 | 4.69E-82 | -1.410596052 | 2.21E-210 | 5.67E-209 |
| 8293 | 'SERF1A' | 8.335390355 | 3.49E-17 | 8.426264755 | 5.51E-18 | 7.60E-18 | 1.03E-18 | 7.876113135 | 1.44E-26 | 6.73E-26 |
| 8317 | 'CDC7' | -1.591738911 | 2.26E-78 | -1.08064578 | 4.83E-24 | 1.43E-79 | 7.33E-25 | -1.379376638 | 4.96E-99 | 6.36E-98 |
| 8318 | 'CDC45' | -1.936269467 | 3.57E-205 | -2.024316428 | 7.3381E-319 | 9.26E-207 | 8.26E-321 | -2.00265984 | 0 | 0 |
| 8345 | 'HIST1H2BH' | -2.742202243 | 1.81E-11 | -2.918863237 | 2.67E-24 | 5.05E-12 | 4.03E-25 | -2.903426253 | 1.01E-35 | 5.79E-35 |
| 83461 | 'CDCA3' | -2.927500875 | 0 | -2.372444969 | 0 | 0 | 0 | -2.587210039 | 0 | 0 |
| 83463 | 'MXD3' | -2.266387841 | 9.45E-89 | -1.935900306 | 3.28E-85 | 5.38E-90 | 1.68E-86 | -1.986376168 | 7.67E-174 | 1.62E-172 |
| 83540 | 'NUF2' | -3.016889014 | 4.27E-107 | -2.137503524 | 5.01E-44 | 2.05E-108 | 4.59E-45 | -2.630308662 | 2.44E-147 | 4.45E-146 |
| 83666 | 'PARP9' | 1.136944719 | 1.68E-89 | 1.301949803 | 1.42E-63 | 9.44E-91 | 9.39E-65 | 1.068657027 | 2.46E-154 | 4.70E-153 |
| 83719 | 'YPEL3' | 1.125077133 | 1.12E-25 | 1.09849972 | 1.23E-18 | 1.81E-26 | 2.23E-19 | 1.087359049 | 1.95E-44 | 1.32E-43 |
| 83879 | 'CDCA7' | -2.593062999 | 4.50E-106 | -1.270961322 | 5.63E-122 | 2.17E-107 | 1.88E-123 | -1.513730108 | 1.62E-211 | 4.21E-210 |
| 84057 | 'MND1' | -1.948774677 | 4.33E-30 | -1.8696628 | 1.31E-22 | 6.23E-31 | 2.06E-23 | -1.903525343 | 1.21E-52 | 9.35E-52 |
| 84171 | 'LOXL4' | 1.46140548 | 2.97E-266 | 1.966833136 | 1.89E-14 | 5.83E-268 | 4.07E-15 | 1.481067698 | 4.74E-286 | 1.69E-284 |
| 84225 | 'ZMYND15' | 1.889817082 | 1.26E-04 | 1.394278939 | 4.35E-04 | 5.46E-05 | 1.84E-04 | 1.547974799 | 3.51E-08 | 8.57E-08 |
| 84258 | 'SYT3' | 1.319124022 | 3.25E-21 | 1.654004145 | 1.19E-07 | 6.06E-22 | 3.73E-08 | 1.319915307 | 8.96E-29 | 4.42E-28 |
| 84419 | 'C15orf48' | 1.302313565 | 2.51E-217 | 1.185836689 | 2.60E-13 | 6.21E-219 | 5.91E-14 | 1.283222663 | 9.29E-236 | 2.70E-234 |
| 84515 | 'MCM8' | -1.37805766 | 8.55E-118 | -1.021826362 | 1.84E-37 | 3.84E-119 | 1.95E-38 | -1.15159803 | 2.12E-148 | 3.91E-147 |
| 84722 | 'PSRC1' | -1.731552098 | 2.28E-117 | -1.479697391 | 2.97E-81 | 1.03E-118 | 1.58E-82 | -1.620688754 | 4.11E-198 | 9.99E-197 |
| 8479 | 'HIRIP3' | -1.544650237 | 8.76E-66 | -1.458646051 | 2.21E-72 | 6.49E-67 | 1.31E-73 | -1.498578118 | 1.27E-138 | 2.19E-137 |
| 84875 | 'PARP10' | 1.066079048 | 1.21E-189 | 2.196691911 | 5.30E-51 | 3.47E-191 | 4.29E-52 | 1.145365302 | 2.08E-235 | 6.05E-234 |
| 84904 | 'ARHGEF39' | -2.481457925 | 7.38E-132 | -1.24348483 | 6.54E-38 | 2.97E-133 | 6.84E-39 | -1.598071714 | 5.23E-154 | 9.96E-153 |
| 84986 | 'ARHGAP19' | -1.459431619 | 2.98E-54 | -1.033015057 | 5.64E-29 | 2.64E-55 | 7.44E-30 | -1.239148627 | 2.83E-81 | 3.11E-80 |
| 8519 | 'IFITM1' | 4.458760598 | 0 | 3.916144605 | 1.33E-147 | 0 | 3.68E-149 | 4.255669285 | 0 | 0 |
| 8520 | 'HAT1' | -1.036322583 | 5.14E-74 | -1.039312924 | 1.68E-36 | 3.41E-75 | 1.82E-37 | -1.042753788 | 6.05E-110 | 8.45E-109 |
| 85366 | 'MYLK2' | -1.833990049 | 3.23E-07 | -2.75802721 | 3.25E-15 | 1.14E-07 | 6.73E-16 | -2.28513787 | 9.99E-22 | 4.11E-21 |
| 8542 | 'APOL1' | 1.958184399 | 0 | 1.641918386 | 1.34E-31 | 0 | 1.66E-32 | 1.948415312 | 0 | 0 |
| 86 | 'ACTL6A' | -1.241045265 | 4.99E-102 | -1.124459862 | 3.70E-101 | 2.50E-103 | 1.57E-102 | -1.189261792 | 6.49E-204 | 1.61E-202 |
| 8622 | 'PDE8B' | 1.133266531 | 8.26E-05 | 1.03170886 | 1.66E-04 | 3.53E-05 | 6.73E-05 | 1.015679775 | 9.19E-09 | 2.31E-08 |
| 8638 | 'OASL' | 2.780792728 | 0 | 3.810966176 | 1.36E-99 | 0 | 5.91E-101 | 2.798800452 | 0 | 0 |
| 8705 | 'B3GALT4' | 1.042228235 | 5.95E-05 | 1.032567862 | 3.40E-09 | 2.51E-05 | 9.65E-10 | 1.024099672 | 1.20E-13 | 3.75E-13 |
| 8722 | 'CTSF' | 1.049449016 | 3.60E-78 | 1.420185697 | 4.57E-308 | 2.29E-79 | 5.4747399999999 | 1.296017003 | 0 | 0 |
| 8787 | 'RGS9' | 2.561878888 | 7.03E-16 | 1.787067908 | 2.00E-29 | 1.61E-16 | 2.60E-30 | 1.974869178 | 2.29E-43 | 1.52E-42 |

| | | | | | | | | | | |
|---|---|---|---|---|---|---|---|---|---|---|
| 8862 | 'APLN' | -1.219009782 | 5.54E-08 | -1.558873272 | 2.00E-20 | 1.86E-08 | 3.40E-21 | -1.446397517 | 4.37E-28 | 2.12E-27 |
| 890 | 'CCNA2' | -3.140870375 | 0 | -2.509085853 | 0 | 0 | 0 | -2.799670584 | 0 | 0 |
| 8900 | 'CCNA1' | -1.506352666 | 8.25E-11 | -1.559562284 | 1.42E-46 | 2.38E-11 | 1.24E-47 | -1.596339558 | 3.58E-58 | 3.00E-57 |
| 891 | 'CCNB1' | -2.077697161 | 0 | -1.76203815 | 0 | 0 | 0 | -1.896431874 | 0 | 0 |
| 89891 | 'WDR34' | -1.108895568 | 2.38E-214 | -1.420535127 | 0 | 5.99E-216 | 0 | -1.337650119 | 0 | 0 |
| 899 | 'CCNF' | -2.372889509 | 5.78E-306 | -1.860806714 | 3.34E-262 | 9.92E-308 | 4.70E-264 | -2.078129651 | 0 | 0 |
| 89958 | 'SAPCD2' | -2.825877652 | 6.23E-255 | -1.0892026 | 1.26E-161 | 1.28E-256 | 3.15E-163 | -1.440076797 | 0 | 0 |
| 9022 | 'CLIC3' | 1.962495163 | 2.45E-235 | 6.087462841 | 1.33E-04 | 5.50E-237 | 5.35E-05 | 1.973175469 | 5.92E-243 | 1.78E-241 |
| 90381 | 'TICRR' | -2.470142099 | 5.17E-185 | -1.423866259 | 7.81E-79 | 1.52E-186 | 4.29E-80 | -1.885815013 | 1.13E-249 | 3.49E-248 |
| 90417 | 'KNSTRN' | -1.499544392 | 1.25E-131 | -1.503008421 | 6.97E-201 | 5.03E-133 | 1.41E-202 | -1.508346432 | 0 | 0 |
| 9055 | 'PRC1' | -2.353821431 | 0 | -1.147613997 | 2.51E-223 | 0 | 4.48E-225 | -1.703021197 | 0 | 0 |
| 9088 | 'PKMYT1' | -1.795407069 | 2.31E-188 | -1.652551814 | 2.14E-151 | 6.68E-190 | 5.81E-153 | -1.727102089 | 0 | 0 |
| 9126 | 'SMC3' | -1.589032165 | 5.93E-100 | -1.335603032 | 5.16E-39 | 3.04E-101 | 5.25E-40 | -1.49773045 | 8.39E-138 | 1.45E-136 |
| 9133 | 'CCNB2' | -2.219823062 | 0 | -1.207175785 | 4.1856672896 | 0 | 4.93634E-316 | -1.507814819 | 0 | 0 |
| 9134 | 'CCNE2' | -4.739848103 | 2.15E-132 | -2.412598454 | 3.38E-40 | 8.65E-134 | 3.36E-41 | -3.392855429 | 9.23E-159 | 1.81E-157 |
| 9149 | 'DYRK1B' | 1.019522558 | 8.67E-43 | 1.568462949 | 0 | 9.51E-44 | 0 | 1.456185628 | 0 | 0 |
| 9156 | 'EXO1' | -2.550610996 | 2.25E-215 | -1.905651699 | 1.06E-73 | 5.67E-217 | 6.15E-75 | -2.350279824 | 3.21E-281 | 1.12E-279 |
| 91801 | 'ALKBH8' | -1.383128251 | 4.14E-25 | -1.093109404 | 1.18E-08 | 6.79E-26 | 3.45E-09 | -1.288648933 | 6.93E-33 | 3.74E-32 |
| 9212 | 'AURKB' | -2.959796458 | 0 | -2.199361453 | 0 | 0 | 0 | -2.489593528 | 0 | 0 |
| 9232 | 'PTTG1' | -2.785968069 | 0 | -1.795553386 | 0 | 0 | 0 | -2.183654494 | 0 | 0 |
| 9252 | 'RPS6KA5' | -2.5360529 | 6.40E-12 | -1.708951218 | 2.41E-08 | 1.75E-12 | 7.19E-09 | -1.69114781 | 1.57E-19 | 6.05E-19 |
| 94030 | 'LRRC4B' | 1.24869949 | 4.37E-33 | 1.131244533 | 8.33E-12 | 5.84E-34 | 2.04E-12 | 1.207764078 | 3.45E-45 | 2.35E-44 |
| 9493 | 'KIF23' | -1.968381396 | 0 | -1.705536164 | 6.13E-203 | 0 | 1.23E-204 | -1.881505134 | 0 | 0 |
| 9586 | 'CREB5' | -1.155278225 | 5.58E-06 | -1.192645078 | 4.42E-04 | 2.17E-06 | 1.88E-04 | -0.98316868 | 1.63E-09 | 4.27E-09 |
| 9636 | 'ISG15' | 2.194956233 | 0 | 1.811599555 | 0 | 0 | 0 | 2.052505085 | 0 | 0 |
| 9700 | 'ESPL1' | -2.920391031 | 0 | -2.279052589 | 0 | 0 | 0 | -2.544800513 | 0 | 0 |
| 9735 | 'KNTC1' | -1.537110982 | 9.73E-93 | -1.83541884 | 1.79E-85 | 5.33E-94 | 9.17E-87 | -1.693697159 | 4.04E-178 | 8.75E-177 |
| 9738 | 'CCP110' | -1.528867939 | 9.81E-82 | -1.100800641 | 2.22E-12 | 5.98E-83 | 5.29E-13 | -1.436855253 | 6.07E-92 | 7.28E-91 |
| 9768 | 'PCLAF' | -3.562011093 | 0 | -2.099013998 | 1.62E-277 | 0 | 2.14E-279 | -2.595348851 | 0 | 0 |
| 9787 | 'DLGAP5' | -2.860931061 | 1.22E-226 | -1.882643049 | 2.26E-66 | 2.87E-228 | 1.43E-67 | -2.466449731 | 6.71E-284 | 2.37E-282 |
| 9824 | 'ARHGAP11A' | -2.430839355 | 2.26E-289 | -1.646102796 | 1.31E-126 | 3.96E-291 | 4.25E-128 | -2.056308329 | 0 | 0 |
| 983 | 'CDK1' | -2.614647596 | 1.89E-230 | -2.059734665 | 1.07E-255 | 4.39E-232 | 1.60E-257 | -2.267282505 | 0 | 0 |
| 9833 | 'MELK' | -1.14352003 | 2.42E-110 | -1.073668316 | 2.45E-109 | 1.13E-111 | 9.45E-111 | -1.123081112 | 1.24E-220 | 3.36E-219 |
| 9837 | 'GINS1' | -1.581765746 | 2.74E-165 | -1.485758138 | 3.86E-59 | 8.87E-167 | 2.73E-60 | -1.550969932 | 4.48E-223 | 1.22E-221 |
| 990 | 'CDC6' | -1.276822495 | 2.35E-154 | -1.48325899 | 3.37E-116 | 8.17E-156 | 1.20E-117 | -1.351469681 | 1.85E-268 | 6.14E-267 |
| 9902 | 'MRC2' | 1.151193143 | 0 | 1.314494699 | 6.16E-235 | 0 | 1.03E-236 | 1.198390362 | 0 | 0 |
| 991 | 'CDC20' | -1.875198673 | 0 | -1.551610861 | 0 | 0 | 0 | -1.682720753 | 0 | 0 |
| 9918 | 'NCAPD2' | -2.402964667 | 0 | -1.065612171 | 0 | 0 | 0 | -1.441626004 | 0 | 0 |
| 9928 | 'KIF14' | -2.872539091 | 9.72E-87 | -1.853158612 | 2.08E-24 | 5.60E-88 | 3.12E-25 | -2.510432584 | 1.06E-107 | 1.44E-106 |
| 993 | 'CDC25A' | -2.185980597 | 9.57E-157 | -1.728911016 | 5.96E-145 | 3.24E-158 | 1.68E-146 | -1.898572486 | 4.27E-300 | 1.62E-298 |
