# Supplementary material for: Palbociclib Enhances Migration and Invasion of Cancer Cells via Senescence-Associated Secretory Phenotype-Related CCL5 in Non-Small-Cell Lung Cancer
Source: J Oncol. 2022 Sep 27;2022:2260625. doi: 10.1155/2022/2260625 (PMC10175017; doi:10.1155/2022/2260625)
Supplement: Supplementary 4 — Supporting information 4. Supplementary Table 3: KEGG enrichment analysis of DEGs between control and 2 μM palbociclib-treated NSCLC cells. [file 2260625.f4.pdf]

Supplementary Table 3 KEGG enrichment analysis of DEGs between control and treated NSCLC cells

KEGG Pathway Term ID	KEGG Pathway Term Desc	KEGG Pathway Term Level1	KEGG Pathway Term Level2	Term Candidate Gene	Total Candidate Gene Num	Term Gene Num	Total Gene Num	Rich Ratio	P value	Q value
4110	Cell cycle	Cellular Process	Cell growth an	43	781	179	15870	0.24022	1.64E-18	5.27E-16
3030	DNA replication	Genetic Informa	Replication an	17	781	49	15870	0.34694	7.23E-11	1.16E-08
4111	Cell cycle - yeast	Cellular Process	Cell growth an	23	781	101	15870	0.22772	5.05E-10	5.42E-08
4113	Meiosis - yeast	Cellular Process	Cell growth an	17	781	77	15870	0.22078	1.49E-07	1.20E-05
4114	Oocyte meiosis	Cellular Process	Cell growth an	26	781	170	15870	0.15294	2.50E-07	1.61E-05
5322	Systemic lupus erythematosus	Human Disease	Immune diseas	30	781	222	15870	0.13514	4.88E-07	2.62E-05
240	Pyrimidine metabolism	Metabolism	Nucleotide met	20	781	139	15870	0.14388	1.52E-05	6.41E-04
4610	Complement and coagulation cascades	Organismal Sys	Immune systen	18	781	118	15870	0.15254	1.79E-05	6.41E-04
5133	Pertussis	Human Disease	Infectious dise	17	781	107	15870	0.15888	1.77E-05	6.41E-04
4914	Progesterone-mediated oocyte maturation	Organismal Sys	Endocrine syst	20	781	142	15870	0.14085	2.09E-05	6.74E-04
5034	Alcoholism	Human Disease	Substance dep	25	781	208	15870	0.12019	3.37E-05	9.87E-04
3440	Homologous recombination	Genetic Informa	Replication an	11	781	54	15870	0.2037	5.25E-05	0.00141
5166	HTLV-I infection	Human Disease	Infectious dise	41	781	447	15870	0.09172	9.46E-05	0.00234
5150	Staphylococcus aureus infection	Human Disease	Infectious dise	19	781	150	15870	0.12667	1.44E-04	0.00309
5168	Herpes simplex infection	Human Disease	Infectious dise	35	781	367	15870	0.09537	1.43E-04	0.00309
5161	Hepatitis B	Human Disease	Infectious dise	23	781	208	15870	0.11058	2.42E-04	0.00459
5206	MicroRNAs in cancer	Human Disease	Cancers: Overv	26	781	248	15870	0.10484	2.32E-04	0.00459
4218	Cellular senescence	Cellular Process	Cell growth an	28	781	287	15870	0.09756	4.46E-04	0.00798
4713	Circadian entrainment	Organismal Sys	Environmental	15	781	120	15870	0.125	8.04E-04	0.01363
4512	ECM-receptor interaction	Environmental I	Signaling mole	15	781	126	15870	0.11905	0.001332	0.02145
5164	Influenza A	Human Disease	Infectious dise	26	781	282	15870	0.0922	0.001601	0.02455
5144	Malaria	Human Disease	Infectious dise	10	781	68	15870	0.14706	0.001716	0.02511
3430	Mismatch repair	Genetic Informa	Replication an	7	781	37	15870	0.18919	0.001921	0.02689
590	Arachidonic acid metabolism	Metabolism	Lipid metaboli	11	781	82	15870	0.13415	0.002199	0.0295
3460	Fanconi anemia pathway	Genetic Informa	Replication an	11	781	85	15870	0.12941	0.002933	0.03498
4060	Cytokine-cytokine receptor interaction	Environmental I	Signaling mole	31	781	371	15870	0.08356	0.002876	0.03498
4668	TNF signaling pathway	Environmental I	Signal transdu	18	781	177	15870	0.10169	0.002828	0.03498
4724	Glutamatergic synapse	Organismal Sys	Nervous systen	15	781	137	15870	0.10949	0.003055	0.03513
4115	p53 signaling pathway	Cellular Process	Cell growth an	12	781	99	15870	0.12121	0.00336	0.03607
4934	Cushing's syndrome	Human Disease	Endocrine and	20	781	208	15870	0.09615	0.00328	0.03607
4620	Toll-like receptor signaling pathway	Organismal Sys	Immune systen	15	781	141	15870	0.10638	0.00402	0.04045
5142	Chagas disease (American	Human Disease	Infectious dise	15	781	141	15870	0.10638	0.00402	0.04045
4657	IL-17 signaling pathway	Organismal Sys	Immune systen	14	781	129	15870	0.10853	0.004478	0.0437
5162	Measles	Human Disease	Infectious dise	19	781	200	15870	0.095	0.004671	0.04424
4725	Cholinergic synapse	Organismal Sys	Nervous systen	15	781	145	15870	0.10345	0.00522	0.04803
