# Supplementary material for: Palbociclib Enhances Migration and Invasion of Cancer Cells via Senescence-Associated Secretory Phenotype-Related CCL5 in Non-Small-Cell Lung Cancer
Source: J Oncol. 2022 Sep 27;2022:2260625. doi: 10.1155/2022/2260625 (PMC10175017; doi:10.1155/2022/2260625)
Supplement: Supplementary 5 — Supporting information 5. Supplementary Table 4: GSEA analysis in control cells (MSigDB C2). [file 2260625.f5.pdf]

Supplementary Table 4 GSEA analysis in control cells (MSigDB C2)

MSigDB C2(curated) KEGG Term Desc	Size	Original size	ES	NES	NOM p-val	FDR q-val	RANK AT MAX	LEADING EDGE
Cell cycle	123	125	-0.6652	-2.902699	0	0	2577	tags=44%, list=14%, signal=51%
DNA replication	36	36	-0.7701	-2.6694558	0	0	1965	tags=69%, list=11%, signal=78%
Spliceosome	126	126	-0.5791	-2.534143	0	0	5713	tags=69%, list=32%, signal=101%
Homologous recombination	28	28	-0.6997	-2.2454782	0	0	2729	tags=61%, list=15%, signal=72%
Mismatch repair	23	23	-0.7243	-2.2106152	0	0	2343	tags=61%, list=13%, signal=70%
Nucleotide excision repair	44	44	-0.5576	-2.1048038	0	4.07E-04	4902	tags=52%, list=27%, signal=72%
Oocyte meiosis	106	113	-0.4811	-2.0765686	0	4.60E-04	3706	tags=35%, list=21%, signal=44%
RNA degradation	56	57	-0.5424	-2.051096	0	7.11E-04	4857	tags=55%, list=27%, signal=76%
Base excision repair	33	33	-0.5632	-1.9238424	0.002083333	0.004986	2507	tags=39%, list=14%, signal=46%
Pyrimidine metabolism	97	98	-0.4499	-1.9156711	0	0.004487	4641	tags=45%, list=26%, signal=61%
Progesterone-mediated oocy	81	85	-0.4571	-1.86054	0	0.007286	1185	tags=19%, list=7%, signal=20%
p53 signaling pathway	68	68	-0.4458	-1.7816523	0.004739337	0.016519	2028	tags=22%, list=11%, signal=25%
Proteasome	41	44	-0.4745	-1.689846	0.002304148	0.036988	6170	tags=71%, list=35%, signal=108%
