# Supplementary material for: Palbociclib Enhances Migration and Invasion of Cancer Cells via Senescence-Associated Secretory Phenotype-Related CCL5 in Non-Small-Cell Lung Cancer
Source: J Oncol. 2022 Sep 27;2022:2260625. doi: 10.1155/2022/2260625 (PMC10175017; doi:10.1155/2022/2260625)
Supplement: Supplementary 6 — Supporting information 6. Supplementary Table 5: GSEA analysis in treated cells (MSigDB C2). [file 2260625.f6.pdf]

Supplementary Table 5 GSEA analysis in treated cells (MSigDB C2)

NAME	MSigDB C2(curated) KEGG Term Desc	Size (after restricting to dataset)	Original size	ES	NES	NOM p-val	FDR q-val	RANK AT MAX	LEADING EDGE
KEGG_ALLO(	Allograft reject	28	37	0.8189218	2.5527902	0	0	2798	tags=71%, list=16%, signal=85%
KEGG_AUTO	Autoimmune tl	31	52	0.7998611	2.52019	0	0	2570	tags=68%, list=14%, signal=79%
KEGG_GRAF	Graft-versus-ho	31	41	0.7566701	2.457526	0	0	2108	tags=65%, list=12%, signal=73%
KEGG_ASTHI	Asthma	23	30	0.8007066	2.3901098	0	0	2250	tags=65%, list=13%, signal=75%
KEGG_TYPE_	Type I diabetes	34	43	0.7224688	2.3510458	0	0	3126	tags=65%, list=17%, signal=78%
KEGG_COMP	Complement ar	55	69	0.6399591	2.3248096	0	0	2839	tags=47%, list=16%, signal=56%
KEGG_INTES	Intestinal immu	40	48	0.6875681	2.3112772	0	0	2699	tags=50%, list=15%, signal=59%
KEGG_LEISH	Leishmania inf	67	72	0.615727	2.307226	0	0	3136	tags=45%, list=18%, signal=54%
KEGG_CYTO	Cytokine-cytok	217	265	0.4866502	2.1811848	0	0	3551	tags=38%, list=20%, signal=47%
KEGG_VIRAI	Viral myocardi	65	70	0.5805287	2.1396017	0	0	3520	tags=45%, list=20%, signal=55%
KEGG_HEMA	Hematopoietic	72	87	0.5365488	2.0756207	0	8.21E-04	3018	tags=38%, list=17%, signal=45%
KEGG_TOLL_	Toll-like recept	88	102	0.5231017	2.0443163	0	9.45E-04	2950	tags=25%, list=17%, signal=30%
KEGG_CYTO	Cytosolic DNA	42	56	0.5978964	2.031984	0	0.001035848	1239	tags=21%, list=7%, signal=23%
KEGG_HYPEI	Hypertrophic c	78	83	0.5051228	1.940059	0	0.00297192	4395	tags=42%, list=25%, signal=56%
KEGG_ALDO	Aldosterone-re	39	42	0.5481566	1.8749526	0.00375235	0.005558752	2828	tags=33%, list=16%, signal=40%
KEGG_DILAT	Dilated cardior	83	90	0.473916	1.8695589	0	0.005636157	4395	tags=42%, list=25%, signal=56%
KEGG_LYSOS	Lysosome	120	121	0.4473984	1.8578392	0	0.006183667	4567	tags=43%, list=26%, signal=57%
KEGG_ANTIC	Antigen proces	68	88	0.488578	1.8283207	0.00175439	0.008697398	3400	tags=44%, list=19%, signal=54%
KEGG_ECM_	ECM-receptor	79	84	0.4634809	1.776479	0	0.015092495	4785	tags=53%, list=27%, signal=72%
KEGG_CELL_	Cell adhesion r	121	133	0.4317553	1.7751197	0	0.014459884	4010	tags=42%, list=22%, signal=54%
KEGG_CARD	Cardiac muscle	67	73	0.4641305	1.7664949	0	0.014638337	4588	tags=36%, list=26%, signal=48%
KEGG_SYSTI	Systemic lupus	115	137	0.4285323	1.7495385	0	0.015772454	2577	tags=41%, list=14%, signal=47%
KEGG_ARAC	Arachidonic ac	51	58	0.4939885	1.7430947	0	0.01608569	5003	tags=53%, list=28%, signal=73%
KEGG_OTHE	Other glycan d	15	16	0.6575533	1.7429032	0.01446655	0.015415453	3490	tags=47%, list=20%, signal=58%
KEGG_CHEM	Chemokine sig	171	189	0.3817108	1.6765195	0	0.030788003	2850	tags=26%, list=16%, signal=30%
KEGG_JAK_S	Jak-STAT sign	130	155	0.397067	1.6530209	0.00665557	0.036916237	4429	tags=32%, list=25%, signal=43%
KEGG_ABC_	ABC transport	41	44	0.4851597	1.6502737	0.0069808	0.036324553	4099	tags=51%, list=23%, signal=66%
KEGG_ALPH	alpha-Linoleni	16	19	0.6068755	1.6475543	0.01926782	0.035860434	5094	tags=56%, list=28%, signal=79%
KEGG_FOCA	Focal adhesion	195	199	0.3662313	1.6179507	0	0.045723733	4564	tags=33%, list=26%, signal=44%
KEGG_RIG_I	RIG-I-like rece	58	71	0.4314548	1.6042029	0.00714286	0.04984226	2798	tags=21%, list=16%, signal=24%
